# Intramedullary nailing versus sliding hip screw for AO/OTA 31-A2 and 31-A3 trochanteric fractures: a systematic review and meta-analysis of randomized controlled trials

**DOI:** 10.1186/s12891-026-10102-w

**Published:** 2026-06-25

**Authors:** Yunjie Zhang, Cornelia Lützner, Pauline Kaboth, Anna Fuhrmann, Carl Neuerburg, Stefanie Deckert

**Affiliations:** 1 Department of Orthopaedics and Trauma Surgery, Musculoskeletal University Center Munich (MUM), University Hospital, LMU Munich, Munich, 81377 Germany; 2 University Center of Orthopedics, Trauma and Plastic Surgery, University Hospital Carl Gustav Carus, TUD Dresden University of Technology, Dresden, Germany; 3 Center for Evidence-based Healthcare, Faculty of Medicine and University Hospital Carl Gustav Carus, TUD Dresden University of Technology, Dresden, Germany; 4 Quality and Medical Risk Management, University Hospital Carl Gustav Carus, TUD Dresden University of Technology, Dresden, Germany

**Keywords:** Trochanteric fracture, Fracture fixation, Intramedullary, Extramedullary, Meta-analysis

## Abstract

**Background:**

Trochanteric femoral fractures (TFFs) are frequent injuries in older adults, with unstable patterns (AO/OTA 31-A2 and A3) carrying a higher risk of complications. Intramedullary nailing (IMN) and sliding hip screw (SHS) are the two main surgical options. However, guideline recommendations differ, and prior meta-analyses are limited. This study aimed to systematically compare the efficacy and safety of IMN versus SHS in treating adult patients with unstable TFFs based on randomized controlled trials (RCTs).

**Methods:**

MEDLINE, Embase, and CENTRAL (January 2008–March 2025) were searched for eligible RCTs, which included adults with 31-A2 or A3 fractures randomized to IMN or SHS. Primary outcomes were mortality and reoperation. Secondary outcomes included implant failures, nonunion, surgical parameters, and postoperative mobility, pain, and function. Risk of bias (RoB) was assessed using RoB 2, and the certainty of evidence (CoE) with GRADE.

**Results:**

Eighteen RCTs (*n* = 3237 patients) were included. No significant differences were found between IMN and SHS in three-month (low CoE) and 12-month mortality (moderate CoE). Reoperation rates trended higher with SHS, but not significantly (pooled OR = 1.70; 95% CI, 0.97–2.97; low CoE). SHS was associated with higher rates of arthroplasty conversion (pooled OR 1.92; 95% CI 1.00–3.68; low CoE), nonunion (pooled OR 1.93; 95% CI 1.12–3.34; low CoE), and infection (pooled OR 2.20; 95% CI 1.29–3.74; low CoE), while implant failure did not differ significantly (pooled OR 1.35; 95% CI 0.91 to 2.01; low CoE). IMN was associated with higher functional scores, less pain (within three months), and a greater likelihood of regaining pre-fracture mobility (CoE low- moderate).

**Conclusions:**

IMN demonstrated comparable mortality to SHS. Although overall reoperation rates did not differ significantly, IMN was associated with lower odds of arthroplasty conversion, nonunion, and infection, as well as reduced early postoperative pain and improved early postoperative function. However, given the overall low to moderate CoE, these findings should be interpreted with caution. Implant selection should remain individualized, taking into account patient characteristics, fracture morphology, and surgeon experience.

**Supplementary Information:**

The online version contains supplementary material available at 10.1186/s12891-026-10102-w.

## Introduction

Trochanteric fractures of the femur (TFFs) are among the most common trauma-related fractures in the geriatric population. For example, between 2009 and 2019, TFFs had the second-highest fracture incidence in Germany at 1.09 per 1,000 population per year [1]. Along with femoral neck fractures, the global number of proximal hip fractures is expected to reach 4.5 million by the year 2050 [2]. This scenario illustrates the major challenges that these injuries pose for medical treatment, rehabilitation and geriatric care, and thus also their economic impact for healthcare systems. Surgical treatment is considered the standard management of TFFs, as conservative treatment may lead to severe complications due to prolonged immobilization [3].

Determining fracture stability is essential for selecting the appropriate surgical strategy and ensuring successful outcomes. Unstable TFFs (AO classification type 31-A2 and A3) present significant challenges compared to stable TFFs (31-A1) due to the loss of medial (calcar) and lateral wall support. This results in varus collapse and rotational instability [4]. This instability transfers excessive stress to fixation implants, increasing the risk of mechanical failure, cut-out, and nonunion [5].

Intramedullary and extramedullary approaches are two surgical techniques used to fixate TFFs. The sliding hip screw (SHS) represents an extramedullary fixation method whose biomechanical properties, particularly its capacity for dynamic fracture compression, provide advantages over other extramedullary systems such as proximal femoral locking compression plates (PF-LCP) and reverse less invasive stabilization systems (Reverse-LISS). SHS became the first-line treatment of TFFs shortly after its introduction in the 1950s [6]. In recent decades, intramedullary fixation with intramedullary nails (IMN), also known as cephalomedullary nails, such as proximal femoral nails and gamma nails, has gained increasing preference among surgeons as an alternative to SHS for managing TFFs, particularly unstable TFFs [7]. The choice between SHS and IMN for managing unstable TFFs is frequently debated. According to the revised National Institute for Health and Care Excellence (NICE) guidelines from the United Kingdom, SHS is recommended for treating AO/OTA type 31-A1 and 31-A2 fractures, while IMN is advised for type 31-A3 fractures [8]. In contrast, the American Academy of Orthopaedic Surgeons (AAOS) guidelines recommend either SHS or IMN for stable TFFs, while IMN is only recommended for unstable TFFs [9]. A large number of randomized trials comparing IMN and SHS across different fracture types have been published, along with numerous systematic reviews. Nevertheless, it seems difficult to reach a clear consensus on implant selection for unstable TFFs, which may be attributed to limitations in the evidence synthesis.

Two meta-analyses have compared the outcomes of IMN and SHS in treating unstable TFFs, but both show methodological shortcomings that limit the use of the results by their addressees [10, 11]. In more detail, the meta-analysis by Raj et al. [10] included randomized controlled trials (RCTs) (searched up to April 2022) that did not adequately distinguish between unstable and stable TFFs. For example, the RCTs by Matre et al. [12] and Little et al. [13] comprised 20% and 40% of stable TFFs, respectively. This lack of distinction between stable and unstable TFFs could substantially compromise data interpretation accuracy because of their inherently different biomechanical characteristics, as mentioned above. The second meta-analysis by Zeelenberg et al. [11] analyzed both RCTs and observational studies (searched up to September 2022). Although the results were illustrated separately, the interpretation was based on merged results from both study types. The study also failed to include all relevant studies based on their inclusion criteria, resulting in an incomplete evidence synthesis. Additionally, neither Raj et al. [10] nor Zeelenberg et al. [11] considered the potential impact of risk of bias (RoB) in individual studies when pooling the results. Thus, the impact of RoB on the presented pooled effect sizes cannot be appraised.

Given the aforementioned limitations, recommendations for routine care are limited. In order to address these limitations and inform the development of a German clinical practice guideline (CPG) for TFF management, the present systematic review aimed to provide a more precise and up-to-date summary and appraisal of RCTs comparing IMN and SHS in the treatment of unstable TFFs with respect to patient-relevant efficacy and safety outcomes.

## Methods

### Data sources and search strategy

This review was conducted according to the Preferred Reporting Items for Systematic Reviews and Meta-Analysis (PRISMA) [14]. A pre-defined protocol was registered in the prospective register of systematic reviews, PROSPERO (CRD42024511889). MEDLINE and Embase (through the interface OVID), and CENTRAL (Cochrane Library) were searched from January 2008 to March 2025 to retrieve pertinent articles. We confined the publication period to align studies with contemporary practices, centering on the newer generations of intramedullary nails. The search strategy consisted of three groups of words representing the following components of the research question: (1) Population/Condition: AO/OTA 31-A2 and 31-A3 TTF, (2) Intervention: IMN, (3) Comparison: SHS. Additionally, we applied a search filter for RCTs provided by each database. We considered RCTs only to summarize the best possible evidence for our research question. Our search was not limited by language restrictions; however, our search strings were developed for English-language databases only, assuming the availability of at least titles and abstracts to be indexed in the English language. Details of the search strategy are reported in Online Supplement 1. Backward citation tracking was performed by checking the references of eligible studies. Forward citation tracking was performed in the Web of Science database by screening the titles of studies that cited eligible studies. References of studies included in orthopedic practice guidelines were screened [8, 9].

### Eligibility criteria and selection process

All published, peer-reviewed RCTs published between January 2008, and March 2025 were eligible for inclusion. Quasi-randomized studies, cohort studies, case-control studies, case reports, and systematic reviews were excluded. RCTs that recruited adults (≥ 18 years old) with a TFF (AO/OTA 31-A2 and 31-A3) undergoing surgery and compared IMN with SHS were included. RCTs considered mixed samples with at least 80% of participants with 31-A2 and 31-A3 or equivalent were also included.

All outcomes of the core outcome set (COS) for hip fracture studies [15] were selected in advance as critical (Table 1). Using a 9-point Likert scale (1 – low importance, 9 – critical for decision-making), the German guideline development group (consisting of trauma surgeons, anesthetists, geriatricians, radiologists, pain medicine specialists, physical therapists, nurses, and patients) assessed the relevance of additional literature-based outcomes for the development of the corresponding guideline recommendation (evidence to decision-making). Thus, reoperation and different surgical complications were rated as critical (see Table 1). Outcomes regarding surgical outcomes, such as blood loss and length of stay, were rated with a lower importance and therefore were not part of the evidence for decision-making (and rating of certainty of evidence).

**Table 1:** Inclusion and exclusion criteria

	Inclusion criteria	Exclusion criteria
Population	Human adults (at least 18 years old)	Animal experiments or studies with children or adolescents (< 18 years old)
Condition	Intertrochanteric femur fracture (any type of AO/OTA 31-A2 and 31-A3 or equivalence) Mixed samples with at least 80% of participants with 31-A2 and 31-A3 or equivalence	All other fracture types and fractures with pathological etiology
Intervention	Intramedullary nails	All other surgical or non-surgical interventions
Comparison	Sliding hip screws	All other surgical or non-surgical interventions
Outcomes	*Primary*: Mortality * Re-operation (incl. Conversion to hip arthroplasty) ^#^ *Secondary*: PRO: pain *, function *, mobility *, ADL * and HR-QoL * surgical complications ^#^ surgical outcomes ^§^	All other outcomes
Study design	RCTs	Non-RCTs such as observational studies (case report/series, cross-sectional, cohort, case-control) and before-after studies, quasi-randomized studies
Publication types	Published peer-reviewed original articles	Study protocols, conference papers, preprints, letters, comments
Time	January 2008- March 2025	earlier than January 2008
Language	no restrictions	

*Abbreviations*: *ADL* activities of daily living, *HR-QoL* health-related quality of life, *PRO* patient-reported outcomes, *RCTs* randomized controlled trials

* Core Outcome Set (COS) for hip fracture trials [15], ^#^ prioritized by the guideline development group as critical for decision making, ^§^ rated as less important by the guideline development group

Thus, the primary outcomes of interest were early mortality (within 3 months postoperatively) and mortality as well as reoperation for the follow-up period of 12 months. If specific reoperation procedures (e.g., conversion to hip arthroplasty) were reported separately, a further analysis was performed. As secondary outcomes, we assessed surgical complications (failure of fixation, nonunion, cut-out, infection, intra- and postoperative fracture) as well as patient-reported outcomes (pain, function, mobility, activities of daily living, and health-related quality of life). Additionally, we considered surgical outcomes (operating time, blood loss, transfusion, length of stay).

Whenever possible, the follow-up periods, firstly within 3 months after surgery and secondly within or at 12 months postoperatively were analysed.

Table 1 provides an overview of the inclusion and exclusion criteria. After pilot selection process, two reviewers performed study selection independently according to predefined eligibility criteria in a two-step approach. First, two reviewers screened titles and abstracts of identified records. Disagreements between individual judgments were resolved through discussion, and, in cases of doubt, the study was carried over to the full-text screening stage. We then retrieved the full-text articles for all doubtful and potentially eligible records and assessed the eligibility of the remaining records. Disagreements were resolved by discussion or by involving a third reviewer. For the selection process, the Rayyan web app was used [16].

### Data extraction and risk of bias assessment

Data were extracted by one reviewer and verified for accuracy and completeness by a second reviewer. Data were entered into a standardised, piloted data extraction form (Excel spreadsheet). At each step of data extraction, we resolved discrepancies by discussion within the group of review authors.

Information on the following was extracted:

- General study information: Author, year, journal, language, registration number, availability of protocol.
- Study characteristics: Study design (including information on randomization, blinding), recruitment time period, study location.
- Participants: age, sex, categorization of unstable TFF (AO/OTA 31-A2 and 31-A3).
- Intervention: IMN.
- Comparator: SHS.
- Outcomes: time point, outcome definition, outcome measurement instrument, outcome values/events/sample size, effect estimate.
- Financial support and disclosures of conflicts of interest.

If necessary, we sought missing data by contacting the authors of relevant publications.

Considering the already available results of the RoB assessment by Raj et al. [10], a well-trained researcher assessed all RCTs using the Cochrane Risk of Bias Tool (RoB 2) for each study and each relevant study outcome. Uncertainties were resolved by involving a second reviewer. The effect of interest is the effect of assignment at baseline, regardless of whether the interventions are received as intended (the ‘intention-to-treat effect’). We used the RoB 2 Excel tool to conduct the assessment and summarized the overall RoB for each result (outcome) according to RoB 2 guidance.

### Statistical analysis

Firstly, we narratively summarized the findings of the included RCTs. This summary contains findings regarding the effects of IMN and SHS on the analyzed outcomes, as well as RoB assessments of these RCTs.

Secondly, when the underlying assumptions of similarity and homogeneity are met and sufficient data are available, we pooled the results using meta-analyses with the random-effects model. The random-effects model was selected a priori, as included studies were expected to differ with respect to patient populations, intervention protocols, and outcome definitions. The random-effects model is the more appropriate choice when effect sizes are anticipated to vary across studies, as it accounts for both within-study and between-study variance [17] and allows generalization beyond the specific set of included studies. In our study, we employed the restricted maximum likelihood (REML) estimator to estimate the between-study variance. All meta-analyses were conducted using the metafor package (version 4.8.0) in R (version 4.4.2). For continuous outcomes, mean differences (MD) with corresponding 95% confidence intervals (CI) were calculated. For dichotomous outcomes, odds ratios (OR) with corresponding 95% confidence intervals (CI) were calculated. Studies with zero events in both treatment groups were excluded from the dichotomous outcome analyses as odds ratios cannot be estimated for such studies.

In case of a meta-analysis with at least three included trials, we quantified statistical heterogeneity of treatment effects between trials using the I² statistic, which describes the percentage of variability in effect estimates attributable to heterogeneity rather than sampling error (I² > 30% moderate heterogeneity, I² > 75% considerable heterogeneity [18]). Additionally, the Chi² test (Cochran’s Q) was employed to test for heterogeneity, with a p-value < 0.10 indicating significant heterogeneity.

The overall effect was tested using the Z-statistic, with statistical significance set at *p* < 0.05. Results were visualized using forest plots, displaying individual study effect estimates, weights, and the pooled effect estimate with 95% CI. For display purposes, forest plot axes were limited to clinically relevant ranges using axis limits, while maintaining complete calculation of all confidence intervals. Studies with non-estimable odds ratios were included in the forest plot but marked as “Not estimable”. We explored potential causes of heterogeneity by sensitivity and subgroup analyses where possible.

In a meta-analysis with at least 10 trials, we explored potential publication bias by visual inspection of a funnel plot. The plots displayed log odds ratios on the x-axis against their standard errors on the y-axis.

### Certainty of evidence

We presented the overall certainty of the evidence (CoE) for each as a critical prioritized outcome (see Table 1) according to the Grading of Recommendations Assessment (GRADE) approach for the domains of RoB, imprecision, indirectness, inconsistency, and other considerations [19], using the guideline tool MAGICapp. The GRADE approach was conducted by the review group and, if needed, revised by the interdisciplinary guideline development group of the German CPG for TFF management. The MAGICapp was used to integrate up- and/or downgrades of the individual domains into an overall judgment of the quality of the body of evidence, resulting in one of four grades: high, moderate, low, or very low. If an outcome was based on one single primary study, the domain ‘inconsistency’ across studies was not applicable, and the overall evaluation was based on the remaining domains. The grading was based on information provided in the (pooled) RCTs.

## Results

### Literature search

**Fig. 1:**
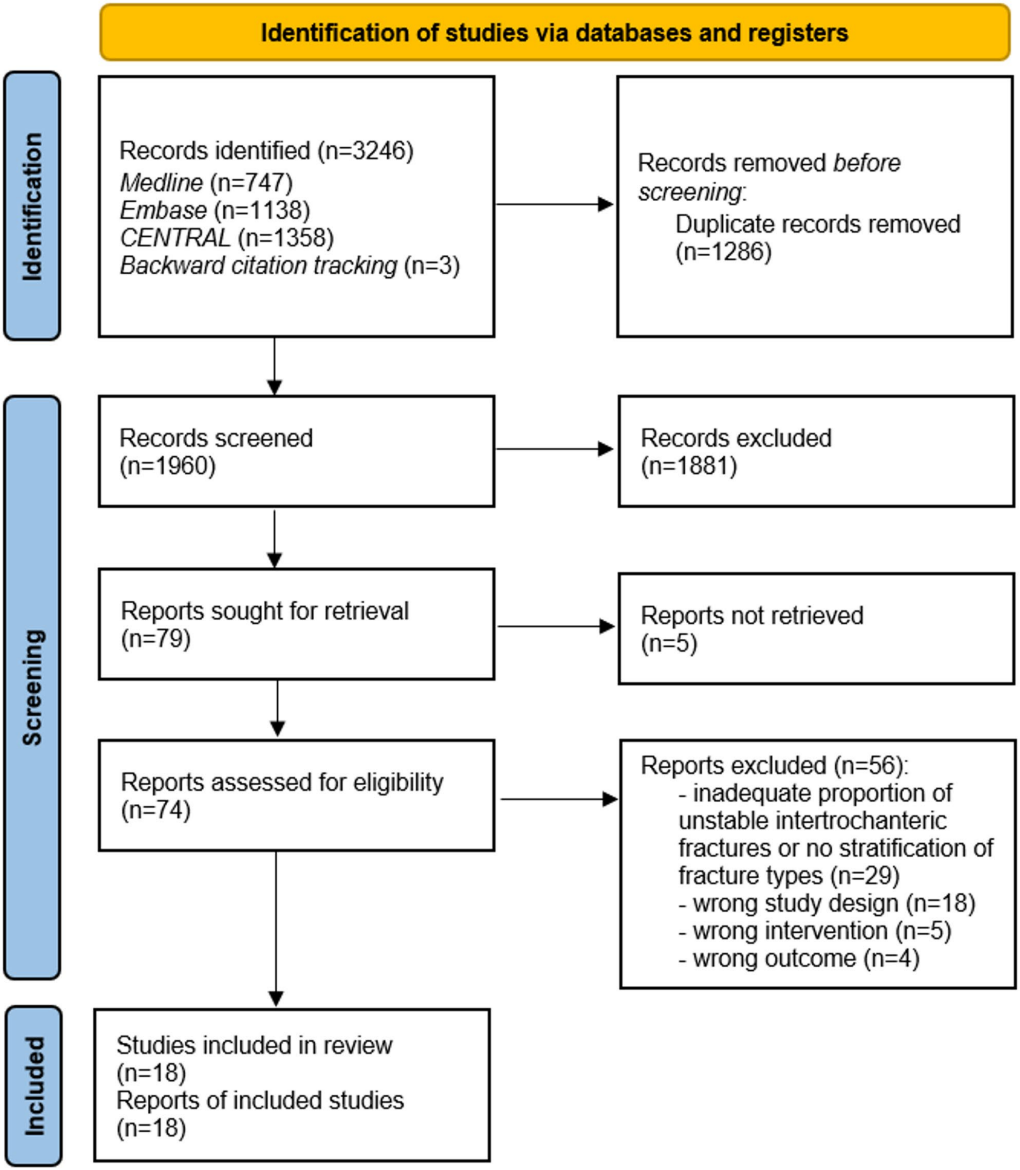
PRISMA flowchart of study selection

The initial search resulted in a total of 3246 records, from which 1286 records were duplicated. After the abstract screening, 74 articles were suitable for full-text retrieval. Following the full-text screening, 18 RCTs were included in this systematic review, one of which was identified by backward citation tracking (Fig. 1). The list of excluded full text is provided in Supplement 2.

### Study characteristics

The 18 RCTs, published from 2009 to 2024, were conducted in multiple countries across Asia, Europe, and North America, including India, the UK, Egypt, Pakistan, Iran, Canada, Colombia, Turkey, Greece, and China (Table 2). A total of 3237 patients were included, 1554 were treated with an IMN, and 1653 with an SHS. According to AO Classification, 2460 had the 31-A2 type fracture, 225 had the 31-A3 fracture, and 179 had either the 31-A2 or 31-A3 fracture (not clearly defined in the original study). A total of 307 cases of 31-A1 type fractures were recorded in three studies [20–22]. The separate illustration of outcomes for 31-A2 fractures was found in 11 studies [7, 21–30]. The outcomes of 31-A3 fractures were only separately provided in the study from Parker et al. [21]; therefore, no separate analyses of A3 fractures could be performed. The mean follow-up time was 11.6 months (10 days to 24 months). Study characteristics are given in Table 2.

**Table 2:** Study characteristics

Author	Year	Country	Fracture types			Sample size		Mean age		Gender (M/F)		Devices	Follow-up
			A1	A2	A3	SHS	IMN	SHS	IMN	SHS	IMN		
Kumar et al. [31]	2024	India	0	N/A	N/A	46	40	47	46	21/25	14/26	DHS vs. PFN	12 m
Kleftouris et al. [7]	2023	UK	0	100%	0%	28	29	84.1	85.7	5/23	6/23	DHS vs. CMN	12 m
Kelany et al. [26]	2023	Egypt	0	100%	0%	15	15	55.5	51.5	4/11	7/8	DHS + TSP vs. PFN	6 m
Kassem et al. [25]	2022	Egypt	0	100%	0%	34	34	68.7	70.8	n.g.	n.g.	DHS + TSP vs. PFN	12 m
Adeel et al. [32]	2020	Pakistan	0	47%	53%	34	34	60.9	59.3	22/12	25/9	DHS vs. PFN	12 m
Andalib et al. [33]	2020	Iran	0	N/A	N/A	55	38	61.5	64.4	26/29	17/21	DHS & DCS vs. CMN	12 m
Das et al. [34]	2020	India	0	52%	48%	75	75	68.8	68.6	27/48	21/54	DHS vs. PFN	24 m
Saleem et al. [35]	2020	Pakistan	0	100%	0%	54	54	60.2	58.5	37/17	31/23	DHS vs. PFN	6 m
Parker et al. [21]	2017	UK	17%^†^	74%	9%	500	500	82.1	82.2	116/384	112/388	SHS vs. PFN	12 m
Sanders et al. [22]	2017	UK, Canada Columbia	17%^†^	83%	0%	126	123	81	80.6	33/93	36/87	SHS vs. PFN	12 m
Reindl et al. [27]	2015	Canada	0	100%	0%	92	112	80	82	31/61	57/55	DHS vs. TFN	12 m
Zehir et al. [30]	2015	Turkey	0	100%	0%	102	96	76.9	77.2	39/63	37/59	DHS vs. PFNA	6 m
Aktselis et al. [23]	2014	Greece	0	100%	0%	35	36	83.1	82.9	7/28	8/28	AMBI hip screw vs. GN	12 m
Bhakat et al. [36]	2013	India	0	57%	43%	30	30	67.8	67.8	13/17	13/17	DHS vs. PFN	12 m
Barton et al. [24]	2010	UK	0	100%	0%	110	100	83.3	83.1	25/85	19/81	SHS vs. long GN	12 m
Verettas et al. [28]	2010	Greece	0	100%	0%	59	59	81	79.2	15/44	20/39	DHS vs. GN	10 d
Xu et al. [29]	2010	China	0	100%	0%	55	51	77.9	78.5	16/39	15/36	DHS vs. PFNA	12 m
Zou et al. [37]	2009	China	78%^††^	22%	0%	63	58	65	65	15/48	12/46	DHS vs. PFNA	12 m

*CMN* Cephalomedullary nail, *DCS* dynamic condylar screw, *DHS* dynamic hip screw, *GN* gamma nail, *IMN* intramedullary nail, *n.g.* not given, *PFN* proximal femoral nail, *SHS* sliding hip screw, *TSP* trochanteric stabilizing plate, *PFNA* proximal femoral nail anti-rotation, *TFN* Trochanteric Fixation Nail

†: Proportion of A1 Type fracture ≤ 20% ††: The data from A1 and A2 type fractures were separately presented, only data from A2 type fractures were extracted and analyzed in the present review

Additionally, details of the intraoperative technical information, such as dynamizing the nail and activating the slide function, as well as the implant length choice, were poorly reported. These factors were not analyzed in the current study.

### Primary outcomes

#### Mortality rate within three months

**Fig. 2:**
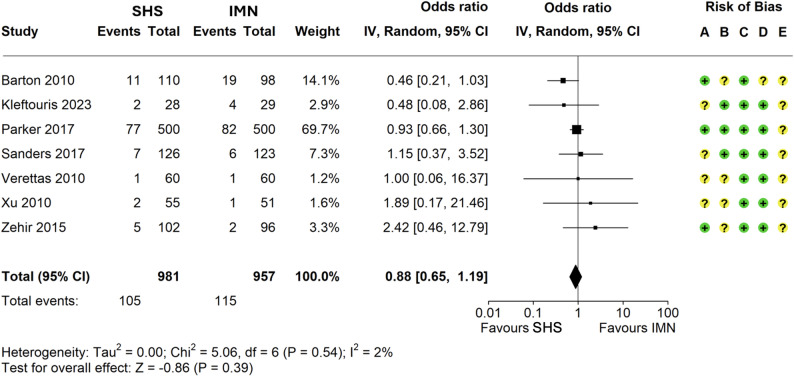
Meta-analysis of mortality rates between SHS and IMN within three months postoperatively and analysis with risk of bias. A: Bias arising from the randomization process, B: Bias due to deviations from the intended interventions, C: Bias due to missing outcome data, D: Bias in measurement of the outcome, E: Bias in selection of the reported result

The early mortality rate within the first three months following treatment for 31-A2 and A3 fractures was reported in seven studies [7, 21, 22, 24, 28–30]. Follow-up time points in the included studies were the early postoperative period, at 1 and 3 months. The analysis found no significant difference in early mortality rate between the two operative methods (OR 0.88; 95% CI 0.65 to 1.19, *p* = 0.39, Fig. 2). The CoE was low due to risk of bias and imprecision (see Supplement 3 and 4, Table 3).

**Table 3:** Meta-analysis of surgical complications until 12 months

Surgical complications	*n* of RCTs	References	SHS/event	IMN/event	OR [95% CI]	*p*-value	I² (%)
Implant failure	16	[18, 21–30, 33, 34, 36, 37]	1346 / 66	1322 / 46	1.35 [0.91–2.01]	0.14	0
Cut out	12	[21–24, 27, 29–31, 34–37]	1183 / 28	1176 / 20	1.29 [0.71–2.34]	0.40	0
Nonunion	9	[21, 22, 26, 29, 31–33, 35, 37]	884 / 42	860 / 22	1.93 [1.12–3.34]	0.02*	0
Intraoperative fracture	6	[23, 25, 28, 29, 34, 36]	288 / 3	285 / 14	0.31 [0.10–0.96]	0.04*	0
Postoperative fracture	8	[7, 21–23, 29–31, 37]	903 / 9	891 / 17	0.65 [0.30–1.43]	0.29	0
Infection combined	15	[21, 24–37]	1270 / 54	1247 / 19	2.20 [1.29–3.74]	< 0.001*	0
Deep infection	12	[21, 24, 25, 27–30, 32–35, 37]	1179 / 13	1162 / 2	2.18 [0.88–5.45]	0.09	0
Superficial infection	12	[21, 24, 25, 27–30, 32–35, 37]	1179 / 37	1162 / 15	1.78 [0.97–3.28]	0.06	0

*RCT* randomized control trial, *SHS* sliding hips screw, *IMN* intramedullary nailing, *OR* odd ratio, *CI* confidence interval

* statistically significant

In 31-A2 fractures (five studies), no significant difference was observed (OR 1.19; 95% CI 0.56 to 2.52, *p* = 0.65, Supplement 4 Table 1).

#### Mortality rate at 12 months

**Fig. 3:**
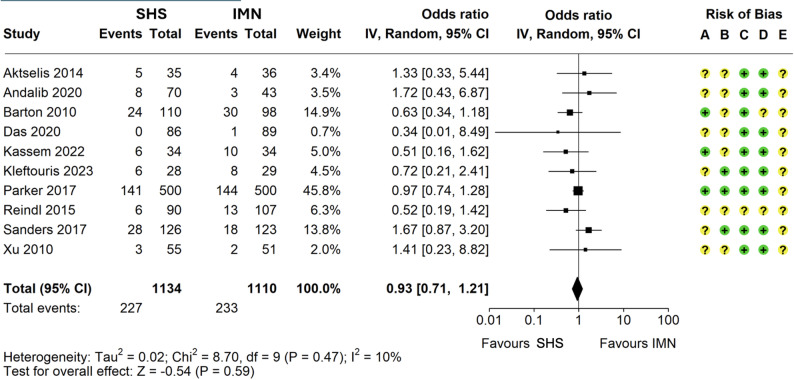
Meta-analysis of mortality rates between SHS and IMN at 12 months postoperatively and analysis with risk of bias. A: Bias arising from the randomization process, B: Bias due to deviations from the intended interventions, C: Bias due to missing outcome data, D: Bias in measurement of the outcome, E: Bias in selection of the reported result

The mortality rates at 12-month follow-up for patients with 31-A2 and A3 fractures were extracted from 10 studies [7, 21–25, 27, 29, 33, 34]. The analysis revealed no significant difference in 12-month mortality between the two operative methods (OR 0.93; 95% CI 0.71 to 1.21, *p* = 0.59, Fig. 3). The CoE was moderate, downgraded for risk of bias (see Supplement 3 and 4, Table 3).

The funnel plot showed a relatively symmetrical distribution around the null effect line, with slight asymmetry suggesting minimal to low publication bias (Supplement 4 Fig. 1a). Studies with negative effect estimates appeared slightly underrepresented.

In the 31-A2 subgroup (seven studies), no significant difference was observed (OR 0.86; 95% CI 0.55 to 1.34, *p* = 0.50, Supplement 4 Table 1).

#### Reoperation rate within 12 months

**Fig. 4:**
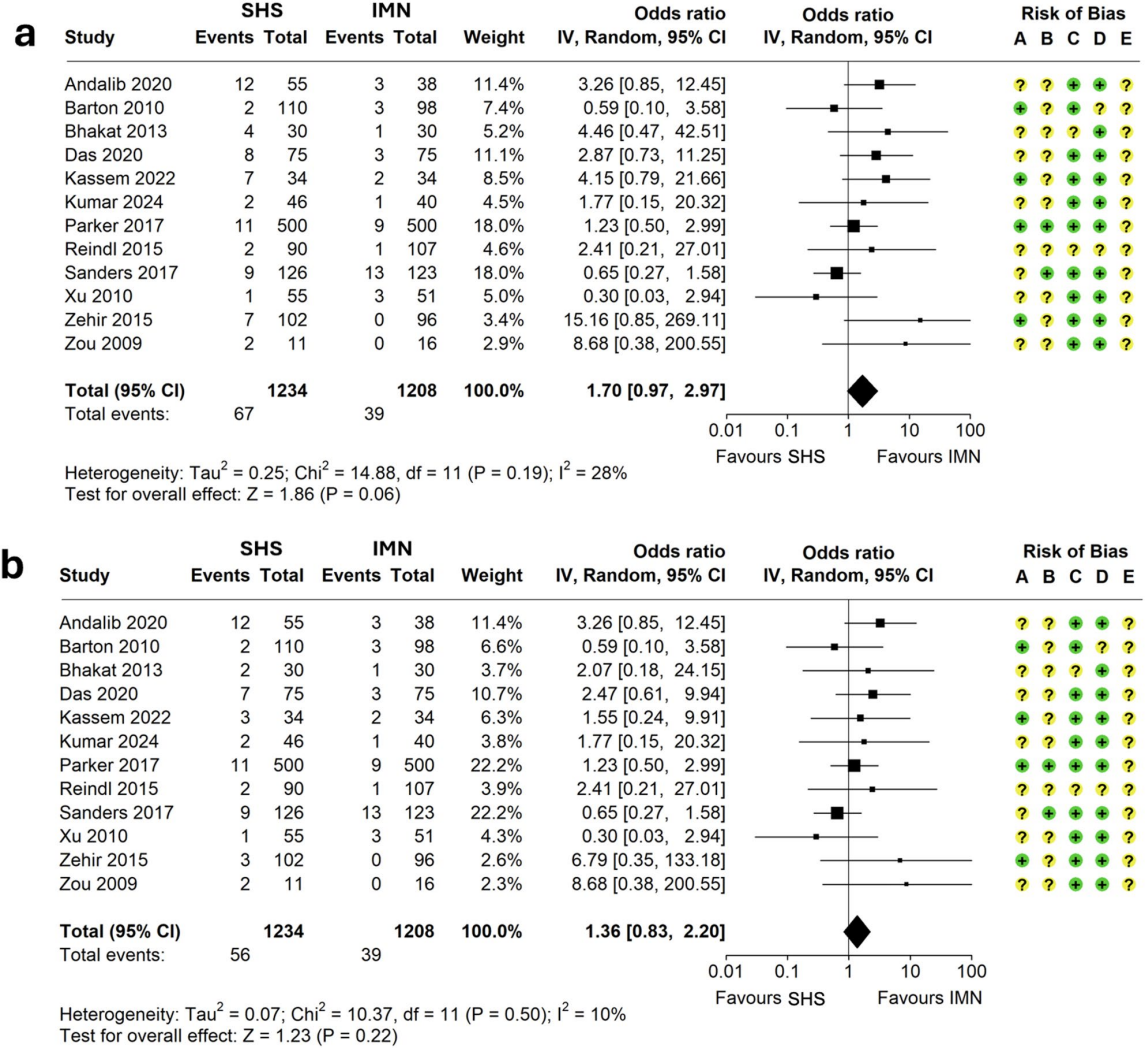
Meta-analysis of reoperation, including debridement (**a**) and without debridement (**b**), after being treated with SHS and IMN, and analysis with risk of bias. A: Bias arising from the randomization process, B: Bias due to deviations from the intended interventions, C: Bias due to missing outcome data, D: Bias in measurement of the outcome, E: Bias in selection of the reported result

Twelve studies [21, 22, 24, 25, 27, 29–31, 33, 34, 36, 37] reported the reoperation rate (any type of surgical procedure) in patients with 31-A2 and A3 fractures. Follow-up time points in the included studies were the early postoperative period, at 6 and 12 months. A trend toward higher reoperation rates was observed with SHS compared with IMN, although this did not reach statistical significance (OR 1.70; 95% CI 0.97 to 2.97, *p* = 0.06; Fig. 4a). The CoE was low due to risk of bias and imprecision (see Supplement 3 and 4, Table 3). In a sensitivity analysis excluding debridement, no differences between groups were observed (OR 1.36; 95% CI 0.83 to 2.20, *p* = 0.22; Fig. 4b).

The funnel plots are depicted in Supplement 4 Fig. 1b and c. Both funnel plots demonstrated noticeable asymmetry, with a concentration of studies on the right side of the null line. This pattern suggests potential publication bias, with possible underreporting of studies showing negative or null effects. The asymmetry was more pronounced for reoperation rates without debridement.

In 31-A2 fractures, no significant differences in reoperation rates were observed, either including debridement (OR 1.27; 95% CI 0.48 to 3.37, *p* = 0.63) or excluding debridement (OR 0.83; 95% CI 0.43 to 1.59, *p* = 0.57) (see Supplement 4 Table 1).

#### Conversion to hip arthroplasty

**Fig. 5:**
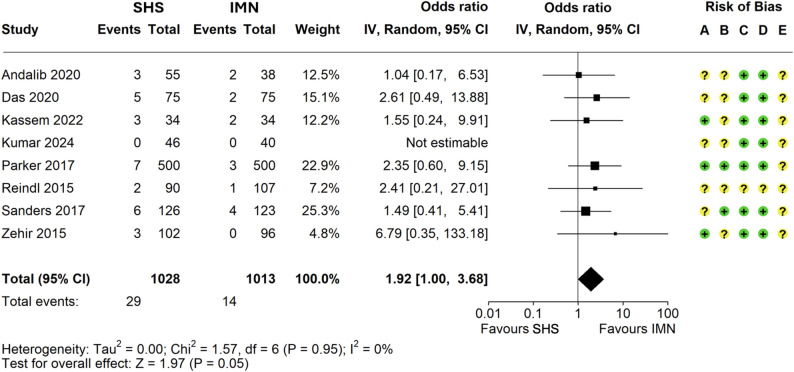
Meta-analysis of conversion to hip arthroplasty between SHS and IMN, and analysis with risk of bias. A: Bias arising from the randomization process, B: Bias due to deviations from the intended interventions, C: Bias due to missing outcome data, D: Bias in measurement of the outcome, E: Bias in selection of the reported result

Pooled analysis of eight studies [21, 22, 25, 27, 30, 31, 33, 34] suggested a possible higher rate of conversion to arthroplasty in patients treated with SHS compared to IMN (OR 1.92; 95% CI 1.00 to 3.68, *p* = 0.05; Fig. 5). Follow-up time points in the included studies were at 6 and 12 months. The CoE was low due to risk of bias and imprecision (see Supplement 3 and 4, Table 3).

In patients with 31-A2 fractures, the analysis did not suggest a higher risk of conversion to arthroplasty (OR 1.74; 95% CI 0.85 to 3.55, *p* = 0.13, Supplement 4 Table 1).

### Secondary outcomes

#### Patient-reported outcomes

Hip pain, various functional outcomes, and recovery of walking ability were reported in the included studies, whereas health-related quality of life or activities of daily living were not reported.

Three studies [23, 30, 34] reported hip pain occurring within three months after surgery. Pooled analysis demonstrated that SHS for unstable TFF was significantly associated with higher odds of early postoperative pain compared with IMN (OR 3.89; 95% CI 1.35 to 11.18, *p* = 0.01) (Supplement 4 Table 2). The CoE was low due to risk of bias, indirectness, and inconsistency (see Supplement 3 and 4, Table 3). In contrast, no significant difference in hip pain was observed between SHS and IMN at 12 months postoperatively (OR 1.74; 95% CI 0.63 to 4.81, *p* = 0.28, Supplement 4 Table 2) [23, 34] with very low CoE due to risk of bias, inconsistency, imprecision (see Supplement 3 and 4, Table 3).

The postoperative function was assessed using the Harris Hip Score (HHS) within three months (follow-up time points were at 1 and 3 months) and at 12 months postoperatively, as reported in two and four studies [25, 32, 34, 36], respectively. SHS was associated with a significantly lower HHS score, with a mean difference of 15.6 points within three months (CoE is very low due to risk of bias (see Supplement 3), inconsistency, and imprecision), and 0.5 points at 12 months postoperatively (CoE is moderate because of risk of bias and inconsistency) (see Supplement 3 and 4, Table 3), as measured on a scale of 0 to 100 (Supplement 4 Table 2). Further function scores, such as the Functional Independence Measure (FIM), Lower Extremity Measure (LEM), and Parker and Palmer Mobility Score (PPMS), were analyzed and reported in Supplement 4 Table 2.

The achievement of recovery to preoperative walking activity (RPWA) within 12 months postoperatively was reported in three studies [25, 29, 30], with follow-up conducted at 6 and 12 months. Pooled analysis demonstrated that SHS was associated with a significantly lower likelihood of regaining preoperative mobility compared with IMN (OR 0.36; 95% CI 0.23 to 0.57, *p* < 0.001) within a year (Supplement 4 Table 2). The CoE was moderate due to risk of bias (see Supplement 3 and 4, Table 3).

### Surgical complications

#### Implant failure

Implant failure was defined as cut-out, Z-effect, reverse Z-effect, plate breakage, proximal screw breakage, cortical screw breakage, and nail breakage. Sixteen studies (median follow-up: 12 months) reported implant failures in 31-A2 and A3 fractures [7, 21–25, 27, 29, 31–34, 36, 37]. No significant difference in implant failure rates was found between IMN and SHS (OR 1.35; 95% CI 0.91 to 2.01, *p* = 0.14, Table 3). The CoE was low due to risk of bias and imprecision (see Supplement 3 and 4, Table 3).

The funnel plot for implant failure showed a reasonably symmetrical distribution with moderate scatter, indicating low risk of publication bias for this outcome (Supplement 4 Fig. 1d).

In the 31-A2 subgroup (10 studies), no significant differences between groups were observed (OR 1.12; 95% CI 0.67 to 1.88, *p* = 0.65, Supplement 4 Table 1).

#### Cut-out

Eleven studies [21–24, 27, 29–31, 34, 36, 37] with a mean follow-up of 12 months reported cut-out rates in 31-A2 and A3 fractures and were included in the meta-analysis. No significant difference was found between IMN and SHS for treating unstable TFF (OR 1.22; 95% CI 0.67 to 2.24, *p* = 0.52, Table 3). The CoE was low due to risk of bias and imprecision (see Supplement 3 and 4, Table 3).

In the 31-A2 subgroup (seven studies), no significant difference in cut-out rates was observed (OR 1.07; 95% CI 0.53 to 2.17, *p* = 0.85, Supplement 4 Table 1).

#### Nonunion

Nonunion was meta-analyzed using nine studies [21, 22, 26, 29, 31–33, 35, 37], with follow-up at 6 and 12 months. Pooled analysis revealed that SHS was associated with a significantly higher likelihood of nonunion compared with IMN in unstable TFF (OR 1.93; 95% CI 1.12 to 3.34, *p* = 0.02, Table 3). The CoE was low due to risk of bias and imprecision (see Supplement 3 and 4, Table 3).

In the 31-A2 subgroup ( five studies), SHS was also associated with a higher likelihood of nonunion (OR 1.99; 95% CI 1.06 to 3.74, *p* = 0.03, Supplement 4 Table 1).

#### Intra- and postoperative fracture and infection

Intra- and postoperative fractures, as well as deep and superficial infections, were meta-analyzed (Table 3) with subgroup results for 31-A2 fractures reported in Supplement 4 Table 1. ). SHS was associated with higher rates of intraoperative fractures and overall infection compared with IMN. The CoE was low due to risk of bias and imprecision (see Supplement 3 and 4, Table 3).

### Surgical outcomes

An overview of meta-analyses for operative time, radiation time, blood loss, and length of hospital stay is summarized in Table 4.

**Table 4:** Meta-analysis of surgical technical parameters

Parameter	*n* of RCTs	references	SHS	IMN	SHS_mean_ - IMN_mean_ [95% CI]	*p*	I² (%)
Operating time (min)	13	[7, 21, 23, 25, 26, 28, 30–32, 34–36]	1067	1053	12.53 [5.77–19.30]	< 0.001*	98.4
Radiation time (sec)	5	[23, 29–31, 34]	313	298	-33.10 [-87.69–21.48]	0.23	99.8
Blood loss (ml)	10	[7, 25, 28–32, 34–36]	517	502	119.5 [71.64 -167.37]	< 0.001*	98.5
Hospital stay (day)	7	[22–24, 28–30, 34]	553	539	0.29 [-1.57 -2.16]	0.67	98.2

*RCT* randomized control trial, *SHS* sliding hips screw, *IMN* intramedullary nailing, *OR* odd ratio, *CI* confidence interval

* statistically significant

The mean operation time was reported in 13 studies [7, 21, 23, 25, 26, 28–32, 34–36], and SHS was associated with a significantly longer operative time compared with IMN, resulting in an average increase of 12.5 min (*p* < 0.001) for unstable TFF fractures.

Five studies reported the mean intraoperative radiation time (in minutes) [23, 29–31, 34] and SHS was associated with an average of 33.1 s less radiation time compared with IMN, although this difference was not statistically significant (*p* = 0.23).

Intraoperative blood loss (ml) was reported in ten studies [7, 25, 28–32, 34–36], with SHS was associated with significantly more blood loss, with an average increase of 119.5 ml (*p* < 0.001).

Mean hospital stay (days) was reported in seven studies [22–24, 28–30, 34], with no significant difference between SHS and IMN (0.29 days shorter for SHS; *p* = 0.67).

### GRADE

Overall, GRADE assessment (summarized in Supplement 4 Table 3) shows that effect estimates comparing SHS and IMN in unstable trochanteric fractures are generally of low to moderate certainty, mainly due to risks of bias, inconsistency, and imprecision .

## Discussion

An increasing use of IMN and a corresponding decline in SHS have been observed in the treatment of TFF over the past two decades. However, the use of SHS is still preferred in low-resource or developing countries for treating intertrochanteric femoral fractures, primarily due to cost, equipment limitations, and surgical familiarity [38]. While several studies [10, 11, 39] have documented intraoperative advantages of IMN, including lower blood loss and shorter operative time, the growing preference for IMN is not yet supported by robust, high-level evidence on functional outcomes, pain, and complications.

The main finding of the present review is that 12-month mortality did not differ significantly between IMN and SHS, in line with other meta-analyses [10, 11, 39]. Despite the relatively large sample size of over 2,000 patients, the CoE was moderate, indicating a reasonable - though not high - level of confidence in this estimate.

IMN was associated with more favorable outcomes than SHS in several aspects of treating unstable TFFs, including lower rates of conversion to hip arthroplasty, nonunion, and overall infection, as well as higher postoperative functional scores. In addition, IMN was associated with shorter operative time and reduced intraoperative blood loss. However, a specific analysis for 31-A3 fractures could not be performed due to insufficient reporting across studies. The certainty of evidence ranged from low to moderate, suggesting that confidence in these estimates is limited and that further research may influence these findings.

Our results suggested 70% higher odds of reoperation with SHS compared with IMN. Although this difference did not reach statistical significance (*p* = 0.06), the low CoE means that we cannot rule out a true effect, and the estimate should therefore be interpreted with caution. In contrast, a recent meta-analysis by Raj et al. [10] reported only a marginal increase (3%) in reoperation rates with SHS in unstable TFFs. This discrepancy may be explained by differences in study selection: although their analysis focused on unstable fractures, the pooled data may have been influenced by the inclusion of stable TFF cases in 9 of the 22 primary studies [12, 13]. In comparison, the present review restricted inclusion to unstable fracture patterns and RCTs, which may provide a more specific estimate of treatment effects. This interpretation is further supported by the low heterogeneity of the pooled estimates.

Notably, the increase in reoperation rates with SHS was reduced to 27% when the analysis was restricted to 31-A2 fractures, compared with the combined analysis of 31-A2 and A3 fractures. This may indicate that a higher reoperation rate is more pronounced in cases with 31-A3 or oblique reverse fractures. Many studies have reported limitations of SHS in the treatment of 31-A3 fractures, as the implant may slide excessively in reverse obliquity patterns, leading to varus collapse and medial displacement of the femoral shaft, particularly in the absence of medial support [40]. Consistent with this, a non-RCT by Park et al. found higher failure rates with SHS in reverse obliquity fractures, including implant cut-out and screw pullout, likely due to increased shear forces compared with IMN [41]. However, due to a lack of adequately reported RCTs, a meta-analysis focusing solely on 31-A3 fractures was not feasible.

Aligning with our findings, one recent meta-analysis by Zeelenberg et al. [11]. compared extramedullary and intramedullary fixation for 31-A2 fractures and reported no significant difference in reoperation rates. However, their analysis included extramedullary devices such as PF-LCP and Reverse-LISS, which differ biomechanically from the SHS. Unlike the SHS, these devices lack dynamic sliding capability and were therefore excluded from the present review. Moreover, Zeelenberg et al. pooled data from both RCTs and observational studies, which may have contributed to increased heterogeneity in their results.

Conversion to hip arthroplasty represents a definitive approach to address the untreatable postoperative complication following a joint-preserving procedure, such as SHS and IMN. Frequent indications are traumatic arthritis, complex implant failure, nonunion or malunion, and avascular necrosis [42]. In our analysis, the odds of arthroplastic conversion were 92% higher with SHS for 31-A2 and A3 combined; but this increase was not confirmed in an analysis of 31-A2 alone. Both estimates showed low heterogeneity. Although SHS are associated with lower initial treatment costs, their higher conversion rates to arthroplasty may offset this economic advantage compared with IMN. Understanding the underlying causes of arthroplasty conversion requires careful evaluation of the spectrum of postoperative complications. Again, these findings need to be viewed with caution due to low CoE.

Specific postoperative complications of higher clinical relevance were prioritized by the guideline development group during CPG development, including cut-out and nonunion. Dealing with a geriatric fracture like TFF, the cut-out of the blade may happen due to osteoporosis [43], which may contribute to the final conversion of hip arthroplasty. Our results demonstrate no difference regarding the cut-out between the two devices.

Another critical factor that may contribute to the conversion of hip arthroplasty is nonunion, especially in unstable TFF [44]. A significantly increased chance of nonunion was found with SHS in the combined 31-A2 and A3 groups (93%) and in the 31-A2 group alone (99%). This might partially explain the aforementioned increased rate of arthroplastic conversion using SHS compared to IMN. In addition to the cut-out, the combined odds of implant failure were also analyzed in the present study, which included Z-effect, reverse Z-effect, plate breakage, screw backout, proximal screw breakage, cortical screw breakage, and Nail breakage. No difference was found between the two devices.

Moreover, infection is also a critical postoperative complication. Our results showed a significant disadvantage of SHS compared with IMN, with 2.2 times higher odds of postoperative infection. This might be due to the open approach of SHS, whereas closed reduction and minimally invasive installation were often used with IMN.

The pooled estimates of surgical complications consistently showed low heterogeneity, suggesting a high degree of comparability across these endpoints. Due to the risk of bias and imprecision, the CoE was rated as low; therefore, the clinical implications of these results should be interpreted with caution.

Last but not least, our data showed that IMN is also associated with less early postoperative pain (within 3 months) and better surgical technical performance, including operating time and blood loss. These advantages might be due to the minimally invasive fashion of the IMN. Nevertheless, findings regarding the combined surgical technical parameters must be interpreted cautiously, as they exhibit high heterogeneity. In terms of postoperative mobility, patients treated with IMN showed better recovery of walking activity within 12 months compared to those treated with SHS and had higher HHS scores within 3 and at 12 months postoperatively. At the individual patient level, it would have been interesting to analyze whether an MCID was achieved when examining established scores; however, this was not reported in the included primary studies. Although the parameters analyzed in the current study are not standard health-related quality-of-life measures, these postoperative functional scores can indirectly reflect quality of life, which should also be considered an important factor in implant selection. The result should be evaluated cautiously in light of the individual situation and the surgeon’s experience.

The present review had several strengths. First, this meta-analysis represents the most up-to-date synthesis of available evidence, with a clear and rigorous distinction between stable and unstable fracture patterns. Second, the prior registration of the study protocol in PROSPERO underscores a commitment to transparency and research integrity. Third, the selection of outcomes was aligned with the internationally recognized core outcome set for hip fracture trials [15] and also corresponded to the rating of the relevance of each outcome by the guideline development group of the German CPG for TFFs management. Fourth, visual inspection of the funnel plots suggested a low to moderate risk of publication bias across outcomes, with the greatest concern for reoperation-related endpoints. Finally, we considered and assessed the RoB at the outcome level for each RCT included and applied the GRADE, a standardized, transparent approach for assessing the certainty of evidence. These methodological approaches ensure a high level of clinical relevance, comparability across the included RCTs, and an adequate, comprehensive approach to assessing the reported results of already published RCTs in this field.

In our study, we employed REML to estimate the between-study variance, overcoming the limitations of the DerSimonian-Laird method, which has been overused despite its susceptibility to bias in small samples [45]. This ensures our pooled estimates are based on a sufficiently reliable calculation of heterogeneity, strengthening the robustness of our conclusions. The further use of small-sample adjustment, such as the Hartung-Knapp adjustment, was not applied to avoid excessively conservative results and to keep the results informative [46–48].

On the contrary, several limitations of the present systematic review should be acknowledged. Although all raw data were carefully screened and only unstable TFFs were included, misclassification of fracture stability within individual studies cannot be completely excluded. In addition, patient characteristics such as age, bone quality, and comorbidities varied across the primary studies, which may have contributed to heterogeneity.

Further limitations include variability in follow-up timing and outcome measures that resulted in limited study numbers for certain endpoints. To generate pooled estimates, follow-up time points that were close in time were grouped (e.g., early postoperative phase, 1 and 3 months).

Regarding publication bias, the relatively small number of included studies (*n* = 10–12 per outcome) limits the interpretability of the funnel plots. The observed asymmetry may reflect factors other than publication bias, including heterogeneity or true small-study effects. Furthermore, variations in surgical technique, surgeon experience, and anesthesia strategies may introduce residual confounding despite attempts to stratify the data.

Another potential source of heterogeneity is the evolution of intramedullary nail design. Early-generation implants, such as the Gamma Nail and Proximal Femoral Nail, were associated with higher complication rates, whereas newer designs, including the Proximal Femoral Nail Antirotation and InterTAN nail, offer improved stability. The included studies were published between 2009 and 2024 (median year 2017), meaning that both early- and newer-generation implants were represented, which may have influenced the pooled results. In addition, residual confounding may persist due to variations in surgeon experience, anesthesia practices, and intraoperative decision-making, which could not be specifically accounted for in our analysis because these details were not consistently reported.

Furthermore, according to the GRADE assessment, the CoE for the primary outcomes ranged from low to moderate (and was very low for some secondary outcomes), indicating that conclusions in favor of a particular treatment must be drawn with caution.

In conclusion, IMN demonstrated mortality rates similar to SHS, but generally appeared to be associated with lower rates of arthroplasty conversion, nonunion, and infection, as well as reduced early postoperative pain and improved early postoperative function in the treatment of TFFs. However, the evidence is limited due to low-to-moderate CoE. Future studies should adhere to reporting guidelines for RCTs [49] and be registered in trial databases to ensure transparency. Implant selection should remain individualized, taking into account patient characteristics, fracture morphology, and surgeon experience.

## Supplementary Information

Supplementary Material 1.

Supplementary Material 2.

Supplementary Material 3.

Supplementary Material 4.

## Data Availability

The datasets used and/or analyzed during the current study are available from the corresponding author on reasonable request.

## Abbreviations

AAOS: American Academy of Orthopaedic Surgeons
ADL: Activites of daily living
AO: Arbeitsgemeinschaft für Osteosynthesefragen
CI: Confidence Interval
CMN: Cephalomedullary Nail
CoE: Certainty of Evidence
COS: Core Outcome Set
CPG: Clinical Practice Guideline
DCS: Dynamic Condylar Screw
FIM: Funktional Independence Measure
GN: Gamma Nail
GRADE: Grading of Recommendations Assessment, Development and Evaluation
HR-QoL: Health-related Quality of Life
IMN: Intramedullary Nail
LEM: Lower Extremity Measure
LISS: Less Invasive Stabilization System
MD: Mean Difference
NICE: National Institute for Health and Care Excellence
OR: Odds Ratios
PF-LCP: Proximal Femoral Locking Compression Plates
PFN: Proximal Femoral Nail
PFNA: Proximal Femoral Nail Anti-Rotation
PPMS: Parker and Palmer Mobility Score
PRIMSA: Preferred Reporting Items for Systematic Reviews
PRO: Patient-reported outcome
RCT: Randomized controlled trial
REML: Restricted maximum likelihood
RoB: The Risk of Bias
RPWA: Recovery to Preoperative Walking Activity
SHS: Sliding hip screw
TFF: Trochanteric Femoral Fracture
TFN: Trochanteric Fixation Nail
TSP: Trochanteric Stabilizing Plate
