# Supplementary material for: Intramedullary nailing versus sliding hip screw for AO/OTA 31-A2 and 31-A3 trochanteric fractures: a systematic review and meta-analysis of randomized controlled trials
Source: BMC Musculoskelet Disord. 2026 Jun 25;27:555. doi: 10.1186/s12891-026-10102-w (PMC13321551; doi:10.1186/s12891-026-10102-w)
Supplement: Supplementary file 1 — Supplementary Material 1. [file 12891_2026_10102_MOESM1_ESM.docx]

**ONLINE SUPPLEMENT 1**

**Search strategy for Medline via Ovid interface**

Last search: 07.03.2025

Search concept: (Intervention OR Comparison) AND Condition AND Design (by search filter)

**Intervention**

1. Intramedullary adj3 nail*.mp.
2. Intra-medullary adj3 nail*.mp.
3. cephalocondylic adj3 nail*.mp.
4. Cephalomedullary adj3 nail*.mp.
5. Cephalo-medullary adj3 nail*.mp.
6. Femor* adj3 nail*.mp.
7. Femur* adj3 nail*.mp.
8. gamma adj3 nail*.mp.
9. exp Fracture Fixation, Internal/
10. internal* adj3 fixat*.mp.
11. intramedullary adj3 fixat*.mp.
12. intra-medullary adj3 fixat*.mp.
13. cephalocondylic adj3 fixat*.mp.
14. Cephalomedullary adj3 fixat*.mp.
15. Cephalo-medullary adj3 fixat*.mp.
16. Femor* adj3 fixat*.mp.
17. Femur* adj3 fixat*.mp.
18. exp bone nails/
19. bone nail*.mp.
20. exp bone plates/
21. bone plate*.mp.
22. or/1-21 (87.577)

**Comparison**

1. exp Bone Screws/
2. bone screw*.mp.
3. (hip and screw).mp.
4. extramedullary adj3 implant*.mp.
5. extra-medullary adj3 implant*.mp.
6. extramedullary adj3 fixat*.mp.
7. extra-medullary adj3 fixat*.mp.
8. external* adj3 fixat*.mp.
9. or/23-30
10. 22 or 31

**Condition**

1. intertrochant*.mp.
2. pertrochant*.mp.
3. per-trochant*.mp.
4. trochant*.mp.
5. stable.mp.
6. unstable.mp.
7. 31-A2.ti,ab.
8. 31-A3.ti,ab.
9. proximal femur*.mp.
10. proximal femor*.mp.
11. extracapsular.mp.
12. extra-capsular.mp.
13. hip.mp.
14. exp hip/
15. exp Hip Fractures/
16. Hip fracture*.mp.
17. or/33-48
18. 32 AND 49
19. limit 50 to (clinical trial or controlled clinical trial or randomized controlled trial)
20. (exp Animals/ or exp Animal Experimentation/ or exp Models, Animal/) not Humans/
21. 51 not 52
22. limit 53 to yr="2008 -Current"
