# Supplementary material for: Intramedullary nailing versus sliding hip screw for AO/OTA 31-A2 and 31-A3 trochanteric fractures: a systematic review and meta-analysis of randomized controlled trials
Source: BMC Musculoskelet Disord. 2026 Jun 25;27:555. doi: 10.1186/s12891-026-10102-w (PMC13321551; doi:10.1186/s12891-026-10102-w)
Supplement: Supplementary file 2 — Supplementary Material 2. [file 12891_2026_10102_MOESM2_ESM.docx]

**ONLINE SUPPLEMENT 2**

**List of excluded full texts with reasons (n=56)**

| **#** | **First author** | **Year** | **Title** | **Reason for exclusion** |
| --- | --- | --- | --- | --- |
| 1 | **Akhtar** | 2016 | Comparison between proximal femoral nail antirotation (PFNA) and dynamic condylar screw (DCS) in the management of unstable proximal femur fractures in term of mean union time | wrong intervention |
| 2 | **Ali Shaikh** | 2023 | Comparison of Operative Parameters during Fixation of Intertrochanteric Fractures with Proximal Femoral Nail and Dynamic Hip Screw | not enough proportion of unstable intertrochanteric fractures or no stratification of fracture types |
| 3 | **Ando** | 2024 | Biomechanical Comparison of Short-, Mid-, and Long-Length Proximal Femoral Nails for Femoral Intertrochanteric Fracture (AO/OTA 31A3.3) Fixation | wrong outcome |
| 4 | **Anjum** | 2021 | Comparison of functional outcome of Proximal Femoral Nail (PFN) and Dynamic Hip Screw (DHS) in intertrochanteric fractures of femur under spinal anesthesia | not enough proportion of unstable intertrochanteric fractures or no stratification of fracture types |
| 5 | **Anshul** | 2022 | To Evaluate the Results of Dynamic Hip Screw (DHS) & Proximal Femoral Nail (PFN) In Intertrochanteric Fractures of Proximal Femur with Special Reference to Surgical Site Infection | not enough proportion of unstable intertrochanteric fractures or no stratification of fracture types |
| 6 | **Barton** | 2012 | A prospective randomised control trial comparing the long gamma nail with the sliding hip screw for the treatment of AO/OTA 31 A2 fractures of the proximal femur | Study design |
| 7 | **Bhakat** | 2013 | Comparitive Study between Proximal Femoral Nailing and Dynamic Hip Screw in Intertrochanteric Fracture of Femur | not enough proportion of unstable intertrochanteric fractures or no stratification of fracture types |
| 8 | **Bretherton** | 2016 | Femoral Medialization, Fixation Failures, and Functional Outcome in Trochanteric Hip Fractures Treated With Either a Sliding Hip Screw or an Intramedullary Nail From Within a Randomized Trial | wrong outcome |
| 9 | **Cai** | 2016 | Comparison of intramedullary and extramedullary fixation of stable intertrochanteric fractures in the elderly: a prospective randomised controlled trial exploring hidden perioperative blood loss | not enough proportion of unstable intertrochanteric fractures or no stratification of fracture types |
| 10 | **Calderón** | 2013 | Proximal femoral intramedullary nail versus DHS plate for the treatment of intertrochanteric fractures. A prospective analysis | wrong study design |
| 11 | **Cao** | 2009 | Dynamic hip screw, Gamma nail and proximal femoral nail in treating intertrochanteric fractures in the elderly: a prospective randomized biocompatibility study of 95 patients | not enough proportion of unstable intertrochanteric fractures or no stratification of fracture types |
| 12 | **Cao** | 2024 | Comparison of external Orthofix pertrochanteric fixator and internal proximal femoral nail antirotation fixation for intertrochanteric fractures in high-risk elderly patients | wrong intervention |
| 13 | **Chauhan** | 2024 | A COMPARATIVE STUDY OF PROXIMAL FEMORAL NAIL AND DYNAMIC HIP SCREW FOR INTERTROCHANTERIC FRACTURES OF THE FEMUR | wrong study design |
| 14 | **Chen** | 2018 | Efficacy of proximal femoral nail anti-rotation and dynamic hip screw internal fixation in the treatment of hip fracture in the elderly patients | not enough proportion of unstable intertrochanteric fractures or no stratification of fracture types |
| 15 | **Claderon** | 2013 | Comparison of proximal femoral intramedullary nail (PFN) versus plate (DHS) to treat intertrochanteric fractures, prospective analysis | wrong study design |
| 16 | **Dubey** | 2021 | Proximal femoral nail (PFN) versus dynamic hip screw (DHS) in unstable intertrochanteric fractures of femur-a comparative clinical study | wrong study design |
| 17 | **Garg** | 2011 | Outcome of short proximal femoral nail antirotation and dynamic hip screw for fixation of unstable trochanteric fractures. A randomised prospective comparative trial | Study design |
| 18 | **Guerra** | 2014 | Functional recovery of elderly patients with surgically-treated intertrochanteric fractures: preliminary results of a randomised trial comparing the dynamic hip screw and proximal femoral nail techniques | not enough proportion of unstable intertrochanteric fractures or no stratification of fracture types |
| 19 | **Guo** | 2013 | Percutaneous compression plate versus proximal femoral nail anti-rotation in treating elderly patients with intertrochanteric fractures: a prospective randomized study | wrong intervention |
| 20 | **Han** | 2021 | Clinical efficacy and safety of PFNA and DHS in the treatment of unstable intertrochanteric fractures in elderly patients | wrong study design |
| 21 | **Harshwardhan** | 2024 | COMPARATIVE ANALYSIS OF FUNCTIONAL OUTCOME OF DYNAMIC HIP SCREW VERSUS PROXIMAL FEMORAL NAILING IN INTERTROCHANTERIC FRACTURES | not enough proportion of unstable intertrochanteric fractures or no stratification of fracture types |
| 22 | **Hazowary** | 2025 | To compare the Efficacy of Proximal Femoral Nailing (PFN) and Dynamic Hip Screw (DHS) Fixation in the Treatment of Intertrochanteric Fractures: a Hospital Based Prospective Study | wrong study design |
| 23 | **Hempel** | 2024 | Single-centre results of a randomised controlled trial comparing the Gamma3 nail and a sliding hip screw to treat AO type 31-A1 and 31-A2 trochanteric fractures | not enough proportion of unstable intertrochanteric fractures or no stratification of fracture types |
| 24 | **Huang** | 2017 | Comparison of the Clinical Effectiveness of PFNA, PFLCP, and DHS in Treatment of Unstable Intertrochanteric Femoral Fracture | wrong intervention |
| 25 | **Jhamnani** | 2024 | Comparative Analysis of Proximal Femoral Nail and Dynamic Hip Screw Fixation in Intertrochanteric Fractures: impact on Stability and Functional Outcomes | not enough proportion of unstable intertrochanteric 26fractures or no str27atification of fract28ure types |
| 26 | **Kesavulu** | 2024 | Comparative Prospective Study on Functional Outcomes of Dynamic Hip Screw Versus Proximal Femoral Nailing for Intertrochanteric Fractures of Femur | wrong study design |
| 27 | **Kunwar** | 2023 | Assessment of Functional Outcome in Intertrochanteric Fractures between Dynamic Hip Screw and Proximal Femoral Nailing: a Comparative Study | wrong study design |
| 28 | **Li** | 2018 | PFNA vs. DHS helical blade for elderly patients with osteoporotic femoral intertrochanteric fractures | wrong study design |
| 29 | **Liang** | 2022 | Proximal femoral nail antirotation versus external fixation for unstable intertrochanteric fractures in elderly patients: a randomized controlled trial | not enough proportion of unstable intertrochanteric fractures or no stratification of fracture types |
| 30 | **Lin** | 2015 | Dynamic hip screw knife system versus spiral blade anti-rotation intramedullary nail in the treatment of unstable intertrochanteric fractures | wrong intervention |
| 31 | **Little** | 2008 | A prospective trial comparing the Holland nail with the dynamic hip screw in the treatment of intertrochanteric fractures of the hip | wrong study design |
| 32 | **Liu** | 2008 | Dynamic hip screw versus Gamma nail in treating intertrochanteric fractures in patients over 70 years: a prospective randomized biocompatibility observation | not enough proportion of unstable intertrochanteric fractures or no stratification of fracture types |
| 33 | **Matre** | 2008 | A prospective randomised multicenter study comparing the sliding hip screw and the Intertan nail in trochanteric and subtrochanteric femoral fractures | not enough proportion of unstable intertrochanteric fractures or no stratification of fracture types |
| 34 | **Matre** | 2012 | Pain, function, and complications after operations with a sliding hip screw or an intertan nail for trochanteric and subtrochanteric fractures. A prospective randomized multiccentre studdy with one year follow-up | not enough proportion of unstable intertrochanteric fractures or no stratification of fracture types |
| 35 | **Matre** | 2013 | Trigen intertan intramedulllary nail versus sliding hip screw. A prospective, randomised multicenter study on pain, function and complications in 684 patients with an intertrochanteric or subtrochanteric fracture and one year follow-up | not enough proportion of unstable intertrochanteric fractures or no stratification of fracture types |
| 36 | **Nakum** | 2024 | A Comparative Study of Functional Outcome of Dynamic Hip Screw Platting Versus Proximal Femoral Nailing in Intertrochanteric Fractures of Femur in Adults | wrong study design |
| 37 | **Ong** | 2019 | Mobility after intertrochanteric hip fracture ﬁ xation with either a sliding hip screw or a cephalomedullary nail: Sub group analysis of a randomised trial of 10 0 0 patients | not enough proportion of unstable intertrochanteric fractures or no stratification of fracture types |
| 38 | **Parker** | 2010 | Intramedullary nails outperformed sliding hip screws in transtrochanteric fracture investigation | wrong study design |
| 39 | **Parker** | 2010 | Randomised trial of extracapsular hip fractures treated with either a sliding hip screw or an intramedullary nail | Study design |
| 40 | **Parker** | 2011 | Intramedullary fixation with a third generation nail versus the sliding hip screw for trohanteric hip fractures; a randomised trial of 400 patients | not enough proportion of unstable intertrochanteric fractures or no stratification of fracture types |
| 41 | **Parker** | 2012 | Sliding hip screw versus the Targon PF nail in the treatment of trochanteric fractures of the hip: a randomised trial of 600 fractures | not enough proportion of unstable intertrochanteric fractures or no stratification of fracture types |
| 42 | **Parker** | 2017 | Sliding hip screw versus the Targon PFT nail for trochanteric hip fractures: a randomised trial of 400 patients | not enough proportion of unstable intertrochanteric fractures or no 38stratification of fra39cture types |
| 43 | **Parmar** | 2024 | A Randomized Prospective study on Comparison between functional and radiological outcome of AO Type 31A1 – A2.1 intertrochanteric fracture fixation by dynamic Hip Screw & PFNA2 | not e40nough proportion of unstable intertrochanteric fractures or no stratification of fracture types |
| 44 | **Sarkar** | 2024 | Comparative Study between Dynamic Hip Screws (DHS) and Plating Proximal Femoral Nailing (PFN) for Trochanteric Fracture of Femur | not enough proportion of unstable intertrochanteric fractures or no stratification of fracture types |
| 45 | **Schemitsch** | 2023 | Intramedullary Nailing vs Sliding Hip Screw in Trochanteric Fracture Management: The INSITE Randomized Clinical Trial | not enough proportion of unstable intertrochanteric fractures or no stratification of fracture types |
| 46 | **Shafi** | 2022 | Comparison between Outcomes of Conventional Dynamic Hip Screws and Proximal Femoral Nail (PFN) Fixation of Intertrochanteric Fracture of the Femur | not enough proportion of unstable intertrochanteric fractures or no stratification of fracture types |
| 47 | **Sharma** | 2015 | Comparison of outcome of management of unstable pertrochanteric femoral fractures with dynamic hip screw and proximal femoral nail | wrong study design |
| 48 | **Sharma** | 2018 | Treatment of stable intertrochanteric fractures of the femur with proximal femoral nail versus dynamic hip screw: a comparative study | wrong study design |
| 49 | **Shen** | 2014 | "Proximal femoral nail versus dynamic hip screw fixation for intertrochanteric |  |
| 50 | **Shyamdhar** | 2023 | A comparative study to evaluate clinical outcome in patients of age above 50 years with intertrochanteric fractures of femur treated with either by PFN (proximal femoral nail) or DHS (dynamic hip screw) | not enough proportion of unstable intertrochanteric fractures or no stratification of fracture types |
| 51 | **Sundararajan** | 2022 | A Comparative Study of Intertrochanteric Fracture Femur Treated with Proximal Femoral Nailing and Dynamic Hip Screw | not enough proportion of unstable intertrochanteric fractures or no stratification of fracture types |
| 52 | **Teng** | 2019 | Computed tomographic image analysis of proximal femoral nail antirotation and dynamic hip screw in the treatment of intertrochanteric fractures | not enough proportion of unstable intertrochanteric fractures or no 49stratification of fra50cture types |
| 53 | **Wang** | 2019 | Comparison of proximal femoral nail antirotation and dynamic hip screw internal fixation on serum markers in elderly patients with intertrochanteric fractures | wrong outcome |
| 54 | **Xu** | 2018 | Comparison of efficacy, complications and TGF-β2 expression between DHS and PFNA in elderly patients with osteoporotic femoral intertrochanteric fracture | wrong outcome |
| 55 | **Yamauchi** | 2014 | Comparison of Functional Recovery in the Very Early Period After Surgery Between Plate and Nail Fixation for Correction of Stable Femoral Intertrochanteric Fractures: a Controlled Clinical Trial of 18 Patients | not enough proportion of unstable intertrochanteric fractures or no stratification of fracture types |
| 56 | **Zhang** | 2017 | Effects of percutaneous compression plate versus proximal femoral nail anti-rotation internal fixation on the postoperative functional recovery and femoral head rotation and nail slippage in elderly patients with stable intertrochanteric fracture | not enough proportion of unstable intertrochanteric fractures or no stratification of fracture types |
