# Supplementary material for: Intramedullary nailing versus sliding hip screw for AO/OTA 31-A2 and 31-A3 trochanteric fractures: a systematic review and meta-analysis of randomized controlled trials
Source: BMC Musculoskelet Disord. 2026 Jun 25;27:555. doi: 10.1186/s12891-026-10102-w (PMC13321551; doi:10.1186/s12891-026-10102-w)
Supplement: Supplementary file 3 — Supplementary Material 3. [file 12891_2026_10102_MOESM3_ESM.docx]

| **Study ID** | | **Outcome** | **Risk of bias** | | | | | |
| --- | --- | --- | --- | --- | --- | --- | --- | --- |
|  |  |  | **D1** | **D2** | **D3** | **D4** | **D5** | **Overall risk of bias** |
| Barton et al. 2010 | | Early Mortality *  (≤ 3 months) | Low | Some concerns | Low | Some concerns | Some concerns | Some concerns |
| Kleftouris et al. 2023 | |  | Some concerns | Low | Low | Low | Some concerns | Some concerns |
| Parker et al. 2017 | |  | Low | Low | Low | Low | Some concerns | Some concerns |
| Sanders et al. 2017 | |  | Some concerns | Low | Low | Low | Some concerns | Some concerns |
| Verettas et al. 2010 | |  | Some concerns | Some concerns | Low | Low | Some concerns | Some concerns |
| Xu et al. 2010 | |  | Some concerns | Some concerns | Low | Low | Some concerns | Some concerns |
| Zehir et al. 2015 | |  | Low | Some concerns | Low | Low | Some concerns | Some concerns |
|  | | | | | | | | |
| Aktselis et al. 2015 | | Mortality *  (12 months) | Some concerns | Some concerns | Low | Low | Some concerns | Some concerns |
| Andalib et al. 2020 | |  | Some concerns | Some concerns | Low | Low | Some concerns | Some concerns |
| Barton et al. 2010 | |  | Low | Some concerns | Low | Some concerns | Some concerns | Some concerns |
| Das et al. 2020 | |  | Some concerns | Some concerns | Low | Low | Some concerns | Some concerns |
| Kassem et al. 2022 | |  | Low | Some concerns | Low | Low | Some concerns | Some concerns |
| Kleftouris et al. 2023 | |  | Some concerns | Low | Low | Low | Some concerns | Some concerns |
| Parker et al. 2017 | |  | Low | Low | Low | Low | Some concerns | Some concerns |
| Reindl et al. 2015 | |  | Some concerns | Some concerns | Some concerns | Some concerns | Some concerns | Some concerns |
| Sanders et al. 2017 | |  | Some concerns | Low | Low | Low | Some concerns | Some concerns |
| Xu et al. 2010 | |  | Some concerns | Some concerns | Low | Low | Some concerns | Some concerns |
|  | | | | | | | | |
| Andalib et al. 2020 | | Reoperation *  (≤ 12 months) | Some concerns | Some concerns | Low | Low | Some concerns | Some concerns |
| Barton et al. 2010 |  |  | Low | Some concerns | Low | Some concerns | Some concerns | Some concerns |
| Bhakat et al. 2013 | |  | Some concerns | Some concerns | Some concerns | Low | Some concerns | Some concerns |
| Das et al. 2020 | |  | Some concerns | Some concerns | Low | Low | Some concerns | Some concerns |
| Kassem et al. 2022 | |  | Low | Some concerns | Low | Low | Some concerns | Some concerns |
| Kumar et al. 2024 | |  | Some concerns | Some concerns | Low | Low | Some concerns | Some concerns |
| Parker et al. 2017 | |  | Low | Low | Low | Low | Some concerns | Some concerns |
| Reindl et al. 2015 | |  | Some concerns | Some concerns | Some concerns | Some concerns | Some concerns | Some concerns |
| Sanders | |  | Some concerns | Low | Low | Low | Some concerns | Some concerns |
| Xu et al. 2010 | |  | Some concerns | Some concerns | Low | Low | Some concerns | Some concerns |
| Zehir et al. 2015 | |  | Low | Some concerns | Low | Low | Some concerns | Some concerns |
| Zou et al. 2009 | |  | Some concerns | Some concerns | Low | Low | Some concerns | Some concerns |
|  | | | | | | | | |
| Andalib et al. 2020 | | Conversion to hip arthroplasty *  (≤ 12 months) | Some concerns | Some concerns | Low | Low | Some concerns | Some concerns |
| Das et al. 2020 | |  | Some concerns | Some concerns | Low | Low | Some concerns | Some concerns |
| Kassem et al. 2020 | |  | Low | Some concerns | Low | Low | Some concerns | Some concerns |
| Kumar et al. 2024 | |  | Some concerns | Some concerns | Low | Low | Some concerns | Some concerns |
| Parker et al. 2017 | |  | Low | Low | Low | Low | Some concerns | Some concerns |
| Reindl et al. 2015 | |  | Some concerns | Some concerns | Some concerns | Some concerns | Some concerns | Some concerns |
| Sanders et al. 2017 | |  | Some concerns | Low | Low | Low | Some concerns | Some concerns |
| Zehir et al. 2015 | |  | Low | Some concerns | Low | Low | Some concerns | Some concerns |
|  | | | | | | | | |
| Adeel et al. 2020 | | Implant failure | Some concerns | Some concerns | Low | Low | Some concerns | Some concerns |
| Aktselis et al. 2014 | |  | Low | Some concerns | Some concerns | Low | Some concerns | Some concerns |
| Andalib et al. 2020 | |  | Some concerns | Some concerns | Low | Low | Some concerns | Some concerns |
| Barton et al. 2010 | |  | Low | Some concerns | Low | Some concerns | Some concerns | Some concerns |
| Bhakat et al. 2013 | |  | Some concerns | Some concerns | Some concerns | Low | Some concerns | Some concerns |
| Das et al. 2020 | |  | Some concerns | Some concerns | Low | Low | Some concerns | Some concerns |
| Kassem et al. 2022 | |  | Low | Some concerns | Low | Low | Some concerns | Some concerns |
| Kelany et al. 2023 | |  | Some concerns | Some concerns | Low | Low | Some concerns | Some concerns |
| Kleftouris et al. 2023 | |  | Some concerns | Low | Low | Low | Some concerns | Some concerns |
| Kumar et al. 2024 | |  | Some concerns | Some concerns | Low | Low | Some concerns | Some concerns |
| Parker et al. 2017 | |  | Low | Low | Low | Low | Some concerns | Some concerns |
| Reindl et al. 2015 | |  | Low | Low | Low | Low | Some concerns | Some concerns |
| Sanders et al. 2017 | |  | Some concerns | Low | Low | Low | Some concerns | Some concerns |
| Xu et al. 2010 | |  | Some concerns | Some concerns | Low | Low | Some concerns | Some concerns |
| Zehir et al. 2015 | |  | Low | Some concerns | Low | Low | Some concerns | Some concerns |
| Zou et al. 2009 | |  | Some concerns | Some concerns | Low | Low | Some concerns | Some concerns |
|  | | | | | | | | |
| Aktselis et al. 2014 | | Cut out | Low | Some concerns | Some concerns | Low | Some concerns | Some concerns |
| Barton et al. 2010 | |  | Low | Some concerns | Low | Some concerns | Some concerns | Some concerns |
| Bhakat et al. 2013 | |  | Some concerns | Some concerns | Some concerns | Low | Some concerns | Some concerns |
| Das et al. 2020 | |  | Some concerns | Some concerns | Low | Low | Some concerns | Some concerns |
| Kumar et al. 2024 | |  | Some concerns | Some concerns | Low | Low | Some concerns | Some concerns |
| Parker et al. 2017 | |  | Low | Low | Low | Low | Some concerns | Some concerns |
| Reindl et al. 2015 | |  | Low | Low | Low | Low | Some concerns | Some concerns |
| Sanders et al. 2017 | |  | Some concerns | Low | Low | Low | Some concerns | Some concerns |
| Xu et al. 2010 | |  | Some concerns | Some concerns | Low | Low | Some concerns | Some concerns |
| Zehir et al. 2015 | |  | Low | Some concerns | Low | Low | Some concerns | Some concerns |
| Zou et al. 2009 | |  | Some concerns | Some concerns | Low | Low | Some concerns | Some concerns |
|  | | | | | | | | |
| Adeel et al. 2020 | | Non-union | Some concerns | Some concerns | Low | Low | Some concerns | Some concerns |
| Andalib et al. 2020 | |  | Some concerns | Some concerns | Low | Low | Some concerns | Some concerns |
| Kelany et al. 2023 | |  | Some concerns | Some concerns | Low | Low | Some concerns | Some concerns |
| Kumar et al. 2024 | |  | Some concerns | Some concerns | Low | Low | Some concerns | Some concerns |
| Parker et al. 2017 | |  | Low | Low | Low | Low | Some concerns | Some concerns |
| Saleem et al. 2020 | |  | Some concerns | Low | Low | Low | Some concerns | Some concerns |
| Sanders et al. 2017 | |  | Some concerns | Low | Low | Low | Some concerns | Some concerns |
| Xu et al. 2010 | |  | Some concerns | Some concerns | Low | Low | Some concerns | Some concerns |
| Zou et al. 2009 | |  | Some concerns | Some concerns | Low | Low | Some concerns | Some concerns |
|  | | | | | | | | |
| Aktselis et al. 2014 | | Length of hospital stay | Low | Some concerns | Some concerns | Low | Some concerns | Some concerns |
| Barton et al. 2010 | |  | Low | Some concerns | Low | Some concerns | Some concerns | Some concerns |
| Das et al. 2020 | |  | Some concerns | Some concerns | Low | Low | Some concerns | Some concerns |
| Sanders et al. 2017 | |  | Some concerns | Low | Low | Low | Some concerns | Some concerns |
| Verettas et al. 2010 | |  | Some concerns | Some concerns | Low | Low | Some concerns | Some concerns |
| Xu et al. 2010 | |  | Some concerns | Some concerns | Low | Low | Some concerns | Some concerns |
| Zou et al. 2009 | |  | Some concerns | Some concerns | Low | Low | Some concerns | Some concerns |
|  | | | | | | | | |
| Bhakat et al. 2013 | | Harris Hip Score  (≤ 3 months) | Some concerns | Some concerns | Some concerns | Some concerns | Some concerns | Some concerns |
| Das et al. 2020 | |  | Some concerns | Some concerns | Low | Some concerns | Some concerns | Some concerns |
|  | | | | | | | | |
| Reindl et al. 2015 | | Functional Independence Measure (3 months) | Low | Low | Low | High | Some concerns | High |
| Sanders et al. 2017 | |  | Some concerns | Low | Some concerns | Some concerns | Some concerns | Some concerns |
|  | | | | | | | | |
| Aktselis et al. 2014 | | Pain (12 months) | Low | Some concerns | Low | Some concerns | Some concerns | Some concerns |
| Das et al. 2020 | |  | Some concerns | Some concerns | Low | Some concerns | Some concerns | Some concerns |
|  | | | | | | | | |
| Kassem et al. 2022 | | Recovery to Preoperative Walking Activity  (≤ 12 months) | Low | Some concerns | Low | Low | Some concerns | Some concerns |
| Xu et al. 2010 | |  | Some concerns | Some concerns | Low | Some concerns | Some concerns | Some concerns |
| Zehir et al. 2015 | |  | Low | Some concerns | Low | Some concerns | Some concerns | Some concerns |
