# Supplementary material for: Intramedullary nailing versus sliding hip screw for AO/OTA 31-A2 and 31-A3 trochanteric fractures: a systematic review and meta-analysis of randomized controlled trials
Source: BMC Musculoskelet Disord. 2026 Jun 25;27:555. doi: 10.1186/s12891-026-10102-w (PMC13321551; doi:10.1186/s12891-026-10102-w)
Supplement: Supplementary file 4 — Supplementary Material 4. [file 12891_2026_10102_MOESM4_ESM.docx]

**ONLINE SUPPLEMENT 4**

**Supplement 4 Table 1:** Meta-analysis of subgroup analysis of fracture type 31-A2

| **Parameters** | **n of RCTs** | **references** | **SHS/event** | **IMN/event** | **OR [95% CI]** | **p-value** | **I² (%)** |
| --- | --- | --- | --- | --- | --- | --- | --- |
| Mortality ≤ 3 m | 5 | [1-5] | 461/17 | 361/14 | 1.19 [0.56, 2.52] | 0.65 | 0 |
| Mortality at 12 m | 7 | [1, 2, 4, 6-9] | 478/78 | 476/85 | 0.86 [0.55, 1.34] | 0.50 | 30.05 |
| Reoperation rate ≤ 12 m incl. debridement | 6 | [2, 4, 5, 7-9] | 517/28 | 509/22 | 1.27 [0.48, 3.37] | 0.63 | 42.79 |
| Reoperation rate ≤ 12 m without debridement | 6 | [2, 4, 5, 7-9] | 517/20 | 509/22 | 0.83 [0.43, 1.59] | 0.57 | 0 |
| Conversion to hip arthroplasty ≤ 12 m | 5 | [2, 5, 8-10] | 716/22 | 713/12 | 1.74 [0.85, 3.55] | 0.13 | 0 |
| Implant failure | 10 | [1, 2, 4-11] | 959/37 | 942/31 | 1.12 [0.67, 1.88] | 0.65 | 1.62 |
| Cut-out | 7 | [2, 4-7, 9, 10] | 970/16 | 977/15 | 1.07 [0.53, 2.17] | 0.85 | 0 |
| Non-union | 5 | [2, 4, 10-12] | 602/32 | 585/16 | 1.99 [1.06, 3.74] | 0.03* | 0 |
| Intraoperative fracture | 4 | [3, 4, 6, 8] | 183/2 | 180/8 | 0.39 [0.09, 1.63] | 0.20 | 0 |
| Postoperative fracture | 7 | [1, 2, 4-6, 10, 13] | 756/9 | 728/16 | 0.67 [0.29, 1.52] | 0.34 | 0 |
| Infection combined | 7 | [3-5, 7-9, 12] | 504/27 | 499/7 | 2.92 [1.32, 6.47] | 0.01* | 0 |
| Deep infection | 7 | [3-5, 7-9, 12] | 504/8 | 499/2 | 1.92 [0.61, 6.04] | 0.27 | 0 |
| Superficial infection | 7 | [3-5, 7-9, 12] | 504/19 | 499/5 | 2.34 [0.95, 5.75] | 0.06 | 0 |

RCT: Randomized Control Trial, SHS: Sliding Hip Screw, IMF: Intramedullary Fixation, OR: Odds Ratio, CI: Confidence Interval;

* Statistically significant

**Supplement 4 Table 2:** Meta-analysis of functional scores, pain, and recovery to preoperative walking activity

| **Parameters** | **n of RCTs** | **references** | **SHS** | **IMN** | **SHS_mean_ - IMN_mean_ [95% CI]** | **p-value** | **I² (%)** |
| --- | --- | --- | --- | --- | --- | --- | --- |
| HHS ≤ 3 m | 2 | [14, 15] | 105 | 105 | -15.55 [-24.82, -6.27] | <0.001* | 98.09 |
| HHS at 12m | 4 | [8, 14-16] | 173 | 173 | -0.45 [-0.84, -0.06] | 0.03* | 0 |
| FIM at 3 m | 2 | [2, 9] | 192 | 206 | 0.11 [-5.09, 5.31] | 0.97 | 63.13 |
| FIM at 12 m | 2 | [2, 9] | 171 | 189 | 1.71 [-1.68, 5.10] | 0.32 | 42.60 |
| LEM at 3 m | 4 | [1, 2, 9, 17] | 271 | 264 | -0.39 [-1.69, 0.92] | 0.56 | 9.20 |
| LEM at 12 m | 3 | [2, 9, 17] | 226 | 227 | -1.68 [-5.24, 1.88] | 0.35 | 68.32 |
| PPMS at 3m | 2 | [4, 6] | 85 | 84 | -0.97 [-1.47, -0.48] | <0.001* | 0 |
| PPMS at 12 m | 2 | [4, 6] | 78 | 76 | -0.78 [-1.30, -0.26] | <0.001* | 0 |
| **Parameters** | **n of RCTs** |  | **SHS/event** | **IMN/event** | **OR [95% CI]** | **p** | **I² (%)** |
| Pain ≤ 3 m | 3 | [5, 6, 15] | 212 / 79 | 207 / 38 | 3.89 [1.35, 11.18] | 0.01* | 59.17 |
| Pain at 12 m | 2 | [6, 15] | 110 / 48 | 111 / 38 | 1.74 [0.63, 4.81] | 0.28 | 13.42 |
| RPWA ≤ 12 m | 3 | [4, 5, 8] | 179 / 54 | 170 / 90 | 0.36 [0.23, 0.57] | <0.001* | 0 |

RCT: Randomized Control Trial, SHS: Sliding Hip Screw, IMF: Intramedullary Fixation, OR: Odds Ratio, CI: Confidence Interval, HHS: Harris Hip Score, FIM: Functional Independence Measure, LEM: Lower Extremity Measure, PPOMIS: Patient-reported Outcomes Measurement Information System, RPWA: Recovery to Preoperative Walking Activity;

* Statistically significant

**Supplement 4 Table 3:** Certainty of evidence (CoE) with GRADE for sliding hip screw vs. intramedullary fixation in unstable trochanteric fractures (for the critical outcomes of the COS of hip fracture trials and the prioritized outcomes by the guideline development group)

| **Outcomes** | **Results** | **Number of**  **participants**  **(studies)** | **Anticipated absolute effects (95% CI) *** | | **Certainty of evidence** |
| --- | --- | --- | --- | --- | --- |
|  |  |  | Risk with sliding hip screw | Risk with intramedullary nailing |  |
| **Primary outcomes** | | | | | |
| **Mortality rate**  **≤ 3 months** | Odds ratio: 0.88  (CI 95% 0.65 - 1.19) | 1`938  (7 studies) | **107** per 1`000 | **120** per 1`000 | **Low**  Due to risk of bias of the body of evidence and imprecision. |
|  |  |  | Difference: **13 less per 1`000**  (CI 95% 39 less - 20 more) | |  |
| **Mortality rate**  **at 12 months** | Odds ratio: 0.93  (CI 95% 0.71 - 1.21) | 2`244  (10 studies) | **190** per 1`000 | **201** per 1`000 | **Moderate**  Due to risk of bias. |
|  |  |  | Difference: **11 less per 1`000**  (CI 95% 49 less - 32 more) | |  |
| **Reoperation rate (incl. debridement)**  **≤ 12 months** | Odds ratio: 1.70  (CI 95% 0.97 - 2.97) | 2`442 patients  (12 studies) | **53** per 1`000 | **32** per 1`000 | **Low**  Due to risk of bias and imprecision. |
|  |  |  | Difference: **21 more per 1`000**  (CI 95% 1 less - 57 more) | |  |
| **Reoperation rate (without debridement)**  **≤ 12 months** | Odds ratio: 1.36  (CI 95% 0.83 - 2.20) | 2`442 patients  (12 studies) | **44** per 1`000 | **32** per 1`000 | **Low**  Due to risk of bias and imprecision. |
|  |  |  | Difference: **12 more per 1`000**  (CI 95% 5 less - 38 more) | |  |
| **Conversion to hip arthroplasty**  **≤ 12 months** | Odds ratio: 1.92  (CI 95% 1.00 - 3.68) | 2`041  (8 studies) | **27** per 1`000 | **14** per 1`000 | **Low**  Due to risk of bias and imprecision. |
|  |  |  | Difference: **13 more per 1`000**  (CI 95% 0 less - 36 more) | |  |
| **Secondary outcomes: Patient-reported outcomes** | | | | | |
| **Hip pain**  **≤ 3 months** | Odds ratio: 3.89  (CI 95% 1.35 - 11.18) | 419  (3 studies) | **467** per 1`000 | **184** per 1`000 | **Low**  Due to risk of bias, indirectness, and inconsistency. |
|  |  |  | Difference: **283 more per 1`000**  (CI 95% 49 more - 532 more) | |  |
| **Hip pain**  **at 12 months** | Odds ratio: 1.74  (CI 95% 0.63 - 4.81) | 221  (2 studies) | **475** per 1`000 | **342** per 1`000 | **Very Low**  Due to risk of bias, inconsistency, imprecision. |
|  |  |  | Difference: **133 more per 1`000**  (CI 95% 95 less - 372 more) | |  |
| **Harris Hip Score**  **≤ 3 months** | Scale of 0 to 100: - the higher the better | 210  (2 studies) | **Mean** | **Mean** | **Very Low**  Due to risk of bias, inconsistency, and imprecision. |
|  |  |  | Difference: **MD 15.55 less**  (CI 95% 24.82 less - 6.27 less) | |  |
| **Harris Hip Score**  **at 12 months** | Scale of 0 to 100: - the higher the better | 346  (4 studies) | **Mean** | **Mean** | **Moderate**  Due to risk of bias and inconsistency. |
|  |  |  | Difference: **MD 0.45 less**  (CI 95% 0.84 less - 0.06 less) | |  |
| **Recovery of walking ability**  **≤ 12 months** | Odds ratio: 0.36  (CI 95% 0.23 - 0.57) | 349  (3 studies) | **20** per 100 | **53** per 100 | **Moderate**  Due to risk of bias. |
|  |  |  | Difference: **33 less per 100**  (CI 95% 40 less - 22 less) | |  |
| **Secondary outcomes: Surgical complications** | | | | | |
| **Implant failure** | Odds ratio: 1.35  (CI 95% 0.91 - 2.01) | 2`668  (16 studies) | **47** per 1`000 | **35** per 1`000 | **Low**  Due to risk of bias and imprecision. |
|  |  |  | Difference: **12 more per 1`000**  (CI 95% 3 less - 33 more) | |  |
| **Cut-out** | Odds ratio: 1.22  (CI 95% 0.67 - 2.24) | 2`329  (11 studies) | **21** per 1`000 | **17** per 1`000 | **Low**  Due to risk of bias and imprecision. |
|  |  |  | Difference: **4 more per 1`000**  (CI 95% 6 less - 20 more) | |  |
| **Non-union** | Odds ratio: 1.93  (CI 95% 1.12 - 3.34) | 1`744  (9 studies) | **49** per 1`000 | **26** per 1`000 | **Low**  Due to risk of bias and imprecision. |
|  |  |  | Difference: **23 more per 1`000**  (CI 95% 3 more - 56 more) | |  |
| **Intraoperative fracture** | Odds ratio: 0.31  (CI 95% 0.10 - 0.96) | 573  (6 studies) | **2** per 1`000 | **5** per 1`000 | **Low**  Due to risk of bias and imprecision. |
|  |  |  | Difference: **3 less per 1`000**  (CI 95% 4 less – 0) | |  |
| **Postoperative fracture** | Odds ratio: 0.65  (CI 95% 0.30 - 1.43) | 1`794  (8 studies) | **12** per 1`000 | **19** per 1`000 | **Low**  Due to risk of bias and imprecision. |
|  |  |  | Difference: **7 less per 1`000**  (CI 95% 13 less - 8 more) | |  |
| **Infection combined** | Odds ratio: 2.20  (CI 95% 1.29 – 3.74) | 2`517  (15 studies) | **33** per 1`000 | **15** per 1`000 | **Low**  Due to risk of bias and imprecision. |
|  |  |  | Difference: **18 more per 1`000**  (CI 95% 4 more - 41 more) | |  |
| **Deep infection** | Odds ratio: 2.18  (CI 95% 0.88 - 5.45) | 2`341  (12 studies) | **4** per 1`000 | **2** per 1`000 | **Low**  Due to risk of bias and imprecision. |
|  |  |  | Difference: **2 more per 1`000**  (CI 95% 0 - 9 more) | |  |
| **Superficial infection** | Odds ratio: 1.78  (CI 95% 0.97 - 3.28) | 2`341  (12 studies) | **23** per 1`000 | **13** per 1`000 | **Low**  Due to risk of bias and imprecision. |
|  |  |  | Difference: **10 more per 1`000**  (CI 95% 0 - 30 more) | |  |

*** The risk in the intervention group (and its 95% confidence interval) is based on the assumed risk in the comparison group and the relative effect of the intervention (and its 95% CI).

CI: Confidence Interval, COS: Core Outcome Set, MD: Mean Difference;

GRADE Grading of Recommendations Assessment, Development, and Evaluation:

High: We are very confident that the true effect lies close to that of the estimate of the effect.

Moderate: We are moderately confident in the effect estimate: The true effect is likely to be close to the estimate of the effect, but there is a possibility that it is substantially different.

Low: Our confidence in the effect estimate is limited: The true effect may be substantially different from the estimate of the effect.

Very Low: We have very little confidence in the effect estimate: The true effect is likely to be substantially different from the estimate of effect

**Supplement 4 Figure 1:** Funnel plot for meta-analyses with more than 10 trials


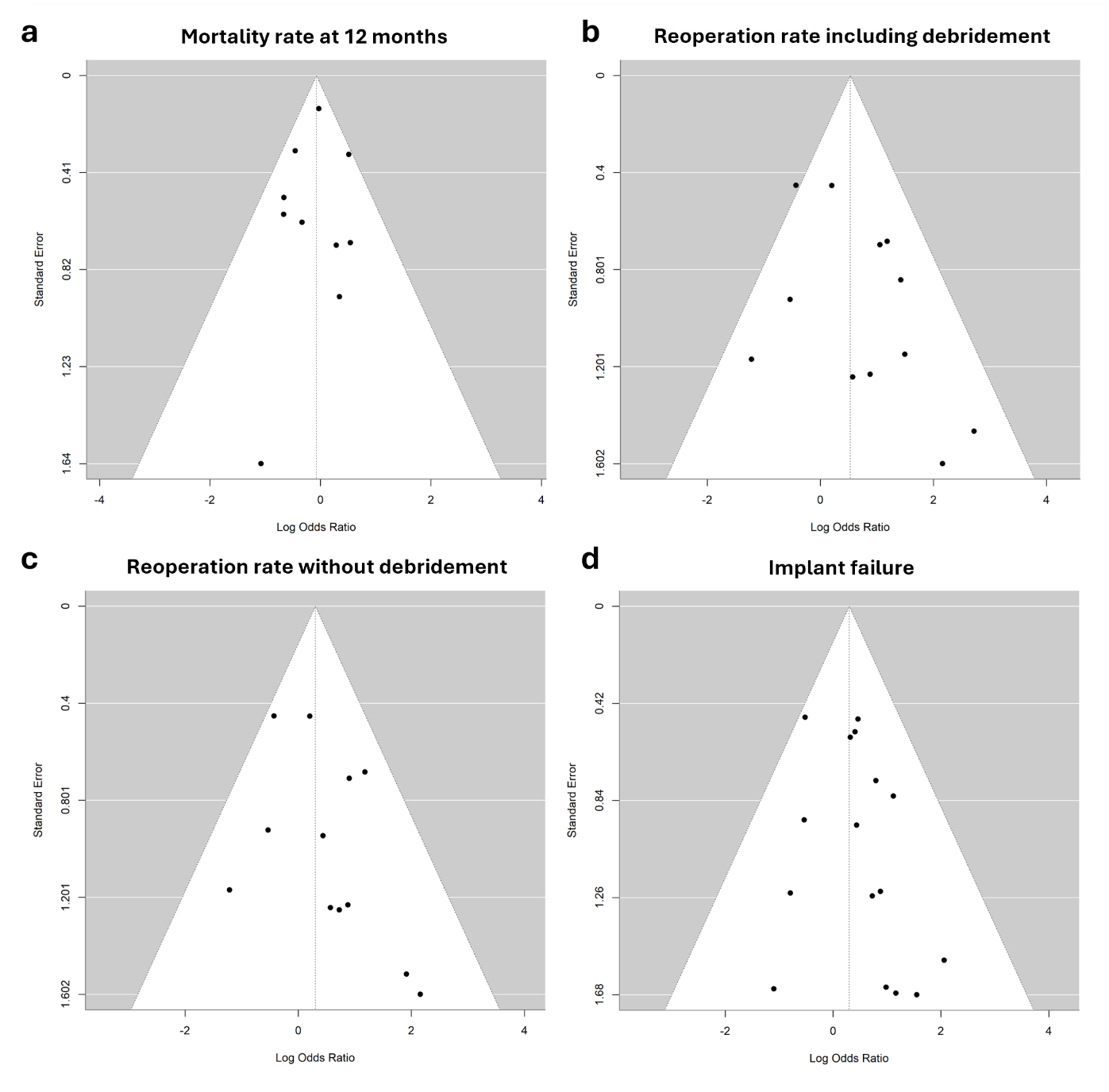


**References:**

1. Kleftouris G, Tosounidis TH, Panteli M, Gathen M, Giannoudis PV: **Endovis Nail versus Dynamic Hip Screw for Unstable Pertrochanteric Fractures: A Feasibility Randomised Control Trial including Patients with Cognitive Impairment**. *J Clin Med* 2023, **12**(13).

2. Sanders D, Bryant D, Tieszer C, Lawendy A-R, MacLeod M, Papp S, Liew A, Viskontas D, Coles C, Gurr K *et al*: **A Multicenter Randomized Control Trial Comparing a Novel Intramedullary Device (InterTAN) Versus Conventional Treatment (Sliding Hip Screw) of Geriatric Hip Fractures**. *Journal of orthopaedic trauma* 2017, **31**(1):1-8.

3. Verettas D-AJ, Ifantidis P, Chatzipapas CN, Drosos GI, Xarchas KC, Chloropoulou P, Kazakos KI, Trypsianis G, Ververidis A: **Systematic effects of surgical treatment of hip fractures: gliding screw-plating vs intramedullary nailing**. *Injury* 2010, **41**(3):279-284.

4. Xu YZ, Geng DC, Mao HQ, Zhu XS, Yang HL: **A comparison of the proximal femoral nail antirotation device and dynamic hip screw in the treatment of unstable pertrochanteric fracture**. *Journal of international medical research* 2010, **38**(4):1266‐1275.

5. Zehir S, Zehir R, Zehir S, Azboy İ, Haykir N: **Proximal femoral nail antirotation against dynamic hip screw for unstable trochanteric fractures; a prospective randomized comparison**. *European journal of trauma and emergency surgery* 2015, **41**(4):393‐400.

6. Aktselis I, Kokoroghiannis C, Fragkomichalos E, Koundis G, Deligeorgis A, Daskalakis E, Vlamis J, Papaioannou N: **Prospective randomised controlled trial of an intramedullary nail versus a sliding hip screw for intertrochanteric fractures of the femur**. *International orthopaedics* 2014, **38**(1):155‐161.

7. Barton TM, Gleeson R, Topliss C, Greenwood R, Harries WJ, Chesser TJ: **A comparison of the long gamma nail with the sliding hip screw for the treatment of AO/OTA 31-A2 fractures of the proximal part of the femur: a prospective randomized trial**. *Journal of bone and joint surgery American volume* 2010, **92**(4):792‐798.

8. Kassem E, Younan R, Abaskhron M, Abo-Elsoud M: **Functional and radiological outcomes of dynamic hip screw with trochanteric stabilizing plate versus short proximal femoral nail in management of unstable trochanteric fractures: a randomized-controlled trial**. *Joint diseases and related surgery* 2022, **33**(3):531‐537.

9. Reindl R, Harvey EJ, Berry GK, Rahme E: **Intramedullary Versus Extramedullary Fixation for Unstable Intertrochanteric Fractures: a Prospective Randomized Controlled Trial**. *Journal of bone and joint surgery American volume* 2015, **97**(23):1905‐1912.

10. Parker MJ: **Sliding hip screw versus intramedullary nail for trochanteric hip fractures; a randomised trial of 1000 patients with presentation of results related to fracture stability**. *Injury* 2017, **48**(12):2762‐2767.

11. Kelany AA, Saied MM, Wahd YESH, Elfeshawy MR: **Dynamic Hip Screw Augmented by Trochanteric Stabilizing Plate versus Proximal Femoral Nail in Fixing Unstable intertrochanteric Femoral Fracture**. *Journal of Population Therapeutics and Clinical Pharmacology* 2023, **30**(4):e34-e44.

12. Saleem M, Ahmed M, Kumar M, Sahar K, Hussain G, Bux M: **Comparison of unstable inter-trochanteric femur fracture treated with dynamic hip screw and proximal femur nail**. *Rawal medical journal* 2020, **45**(3):648‐651.

13. Kumar A, Ahmad W, Kumar S: **Comparison of intramedullary and extramedullary fixation devices in unstable trochanteric fractures**. *International Journal of Pharmaceutical and Clinical Research* 2024, **16**(6):1819-1824.

14. Bhakat U, Bandyopadhayay R: **Comparitive study between proximal femoral nailing and dynamic hip screw in intertrochanteric fracture of femur**. *Open Journal of Orthopedics* 2013, **3**(07):291.

15. Das PB, Singh A, Lenka BS, Pani S: **Osteosynthesis of intertrochanteric fractures by PFN and DHS - A prospective randomized comparative study**. *Journal of Orthopaedics, Trauma and Rehabilitation* 2020, **2020**.

16. Adeel K, Nadeem RD, Akhtar M, Sah RK, Mohy-Ud-Din I: **Comparison of proximal femoral nail (PFN) and dynamic hip screw (DHS) for the treatment of AO type A2 and A3 pertrochanteric fractures of femur**. *JPMA The Journal of the Pakistan Medical Association* 2020, **70**(5):815‐819.

17. Andalib A, Etemadifar M, Yavari P: **Clinical outcomes of intramedullary and extramedullary fixation in unstable intertrochanteric fractures: a randomized clinical trial**. *The archives of bone and joint surgery* 2020, **8**(2):190‐197.
